# Early and late effects of aspirin and naproxen on microRNAs in the lung and blood of mice, either unexposed or exposed to cigarette smoke

**DOI:** 10.18632/oncotarget.20464

**Published:** 2017-08-24

**Authors:** Alberto Izzotti, Roumen Balansky, Gancho Ganchev, Marietta Iltcheva, Mariagrazia Longobardi, Alessandra Pulliero, Anna Camoirano, Francesco D'Agostini, Marta Geretto, Rosanna T. Micale, Sebastiano La Maestra, Mark Steven Miller, Vernon E. Steele, Silvio De Flora

**Affiliations:** ^1^ Department of Health Sciences, University of Genoa, Genoa, Italy; ^2^ IRCCS AOU San Martino IST, Genoa, Italy; ^3^ National Center of Oncology, Sofia, Bulgaria; ^4^ Chemopreventive Agent Development Research Group, Division of Cancer Prevention, National Cancer Institute, Rockville, MD, USA

**Keywords:** lung microRNA, blood microRNA, lung carcinogenesis, cigarette smoke, anti-inflammatory drugs

## Abstract

We recently showed that nonsteroidal anti-inflammatory drugs (NSAIDs) are able to inhibit the lung tumors induced by cigarette smoke, either mainstream (MCS) or environmental (ECS), in female mice. We used subsets of mice to analyze the expression of 1135 microRNAs in both lung and blood serum, as related to the whole-body exposure to smoke and/or oral administration of either aspirin or naproxen. In a first study, we evaluated early microRNA alterations in A/J mice exposed to ECS for 10 weeks, starting at birth, and/or treated with NSAIDs for 6 weeks, starting after weaning. At that time, when no histopathological change were apparent, ECS caused a considerable downregulation of pulmonary microRNAs affecting both adaptive mechanisms and disease-related pathways. Aspirin and naproxen modulated, with intergender differences, the expression of microRNAs having a variety of functions, also including regulation of cyclooxygenases and inflammation. In a second study, we evaluated late microRNA alterations in Swiss H mice exposed to MCS during the first 4 months of life and treated with NSAIDs after weaning until 7.5 months of life, when tumors were detected in mouse lung. Modulation of pulmonary microRNAs by the two NSAIDs was correlated with their ability to prevent preneoplastic lesions (microadenomas) and adenomas in the lung. In both studies, exposure to smoke and/or treatment with NSAIDs also modulated microRNA profiles in the blood serum. However, their levels were poorly correlated with those of pulmonary microRNAs, presumably because circulating microRNAs reflect the contributions from multiple organs and not only from lung.

## INTRODUCTION

Tobacco smoking has been causally associated with the induction of lung cancer and other cancers affecting the respiratory tract, urinary tract, digestive system, and hematopoietic system [1]. Smoking is also one of the main risk factors for other chronic degenerative diseases [2]. Exposure to cigarette smoke (CS) may occur either actively via mainstream CS (MCS) or involuntarily via environmental CS (ECS) or second-hand smoke. Both MCS and ECS are complex mixtures containing several thousand compounds [3], the combustion of *Nicotiana tabacum* leaves generating more than 5000 identified products, 73 of which have been shown to be carcinogenic to humans and/or experimental animals [4]. Although SCS and MCS share a similar chemical composition, there are quantitative differences due to the higher combustion temperature of MCS [5]. SCS is 4 times more toxic than MCS, and SCS condensates are 2–6 times more tumorigenic than MCS condensates in the mouse skin at equivalent concentrations [6]. However, it should be taken into account that MCS is inhaled into the respiratory tract in concentrated form, while ECS is considerably diluted in indoor air. Nevertheless, the respirable particulate matter in certain entertainment venues has been estimated to range from less than 15 mg/m^3^ where smoking is prohibited up to 350 mg/m^3^ where smoking is allowed. In the home environment, peaks up to 300 mg/m^3^ have been found, and inside vehicles concentrations are estimated to range from about 90 mg/m^3^ to well over 1000 mg/m^3^ [3].

The most obvious strategy for the prevention of cancer and other smoking-related diseases is to avoid exposures either to MCS by refraining from smoking (never smokers) or by quitting smoking (ex-smokers) or to ECS, by minimizing exposures in indoor environments through suitable regulations (passive or involuntary smokers). These strategies have been shown not only to attenuate the epidemics of CS-related lung cancers in the male populations of many countries but also to decrease the prevalence of exposure to ECS [7]. As a complementary strategy, it is possible to mitigate the risk of developing CS-related diseases by rendering the organism more resistant to its noxious components, a goal that can be achieved by favoring the intake of protective dietary and pharmacological agents and by modulating protective mechanisms. Such an approach, referred to as chemoprevention, is particularly targeted to three broad categories in the population, including active smokers who are addicted to nicotine and are unable to quit smoking, passive smokers, and ex-smokers. It is also important to evaluate how the intake of certain foods and drugs used for therapeutic purposes can affect the lung carcinogenesis process in smokers [8].

Cancer chemoprevention can be pursued by exploiting several mechanisms, since in principle any step and pathway involved in the carcinogenesis process can be modulated exogenously [9]. Chronic inflammation is one of the key mechanisms contributing to various stages of carcinogenesis [10, 11] and is crucial in CS-related lung carcinogenesis [12]. Accordingly, an anti-inflammatory approach is particularly promising in the prevention of CS-associated cancers [13]. Also due to the very extensive use in the population, nonsteroidal anti-inflammatory drugs (NSAIDs) are of great interest [14]. The salicylate derivative aspirin, or acetylsalicylic acid, which is the most extensively used drug in the world not only for therapeutic purposes but also for the prevention of cardiovascular diseases thanks to its platelet aggregation inhibiting properties, and the propionic acid derivative naproxen are dual inhibitors of the cyclooxygenases COX-1 and COX-2 sharing anti-inflammatory, antipyretic, and analgesic properties.

The protective role of aspirin and other NSAIDs in humans is well established for colorectal cancer, whereas there are uncertainties regarding lung cancer and other types of cancer [15]. However, a recent pooled analysis convincingly showed a significant attenuation of deaths due to lung cancer, particularly to adenocarcinoma [16]. In experimental animal studies, aspirin inhibited the lung tumors induced either by 4-(methylnitrosamino)-1-(3-pyridyl)-1-butanone (NNK) in A/J mice [17] or by 7,12-dimethylbenz(*a*)anthracene (DMBA) in Balb/c mice [18]. Naproxen exerted some protective effects in rodent models but failed to inhibit NNK-induced lung tumors in mice [17].

Up to recently, NSAIDs had never been tested for the ability to modulate lung carcinogenesis resulting from exposure to CS as a complex mixture rather than to its representative components. We have developed and extensively applied a murine model in which Swiss H mice, originated from Swiss albino mice, are exposed to MCS starting at birth. When the mice are exposed to MCS during the first 4 months of life and thereafter are kept for an additional 3–4 months in filtered air in order to allow a suitable growth of tumors, MCS induces a variety of lesions in the lungs, of either inflammatory or preneoplastic or neoplastic nature, as well as preneoplastic lesions in the urinary tract [19, 20].

We applied the above model to evaluate both safety and efficacy of a number of natural products and pharmacological agents, which were administered according to protocols mimicking interventions either in current smokers or in ex-smokers or even by reproducing a transplacental chemoprevention [reviewed by ref. 8]. Among other agents, we investigated the ability of anti-inflammatory agents, also including aspirin and naproxen. After 4 months of exposure to MCS since birth, naproxen was able to attenuate the MCS-induced systemic genotoxic damage in female mice. After an additional 3.5 months in filtered air, both aspirin and naproxen consistently decreased the incidence of lung adenomas, an effect that was statistically significant in female mice [21]. These findings were confirmed in a separate study in A/J mice exposed to ECS, which followed a protocol similar to the model developed by Hanspeter Witschi [22] and also used in our laboratory [23], in which the lung tumorigenicity is due to gas phase components of ECS [24]. However, we started exposure to ECS at birth, when a tremendous oxidative challenge occurs “physiologically” in the lung thereby causing nucleotide and gene expression alterations [25]. After exposure to ECS for 10 weeks since birth and administration of NSAIDs after weaning, both aspirin and naproxen inhibited the ECS-related formation of bulky DNA adducts and 8-hydroxy-2′-deoxyguanosine in lung. Moreover, after 4 months of exposure to ECS followed by 5 months in filtered air, both NSAIDs attenuated the yield of ECS-induced lung tumors, but prevention of ECS-induced lung adenomas was statistically significant only in female mice treated with aspirin [26].

In order to gain further mechanistic insights, we evaluated microRNA (miRNA) expression profiles in subsets of mice of both genders used in the above cancer chemoprevention studies [21, 26]. Modulation of miRNAs in mice treated with NSAIDs, either unexposed or exposed to CS, was investigated both in lung and in blood serum. The goal of analyzing circulating miRNAs was to evaluate its predictivity as a molecular biomarker for the early detection of CS-related lung carcinogenesis and for the identification of subjects responsive to NSAIDs by using noninvasive techniques applicable to humans. Although the two studies reported in the present paper had a similar methodological approach in the assessment of biomarkers, they were independent and had different goals. In fact, the first one examined the same ECS-exposed A/J mice used in the previous cancer chemoprevention study [26]. This study evaluated, in parallel to the aforementioned nucleotide alterations, early alterations of miRNAs detectable after 10 weeks of exposure to ECS and/or 6 weeks of treatment with NSAIDs, when no histopathological change was still appreciable. The second study examined the same MCS-exposed Swiss H mice used in the previous cancer chemoprevention study [21] and evaluated late alterations of miRNAs detectable after 7.5 months, when preneoplastic lesions and tumors occurred in mouse lung. The results relative to miRNA modulation as related to exposure to MCS and to histopathological alterations in Swiss H mice have previously been published [27]. Here we present the data relative to the effects of aspirin and naproxen either in unexposed mice or in CS-exposed mice.

The results obtained highlight a complex picture underlying the early and late effects of either ECS or MCS and/or modulation by NSAIDs of the miRNA machinery in lung and blood serum. They complement the data relative to other intermediate biomarkers and to occurrence of CS-related lung tumors.

## RESULTS

### Early effects of aspirin and naproxen on miRNA profiles in the lung and blood of A/J mice, either unexposed or exposed to ECS

#### Analysis of pulmonary RNA

Evaluation of the 260/280 and 260/230 ratios by fiber optic spectrophotometry provided evidence for a satisfactory purity of all pulmonary RNA samples (data not shown). The structural integrity was evaluated by calculating the RNA Integrity Number (RIN), which is obtained by calculating the ratio between ribosomal RNA bands and total RNA. The average RIN value was 6.3 ± 0.12 (mean ± SE), which is higher than the usually adopted value of 5.0 for establishing adequate RNA integrity. From a quantitative standpoint, the obtained RNA amounts exceeded in all samples the minimum level required to perform miRNA expression analyses by microarray (1 μg).

#### Microarray analysis of pulmonary miRNAs

**Figure 1:**
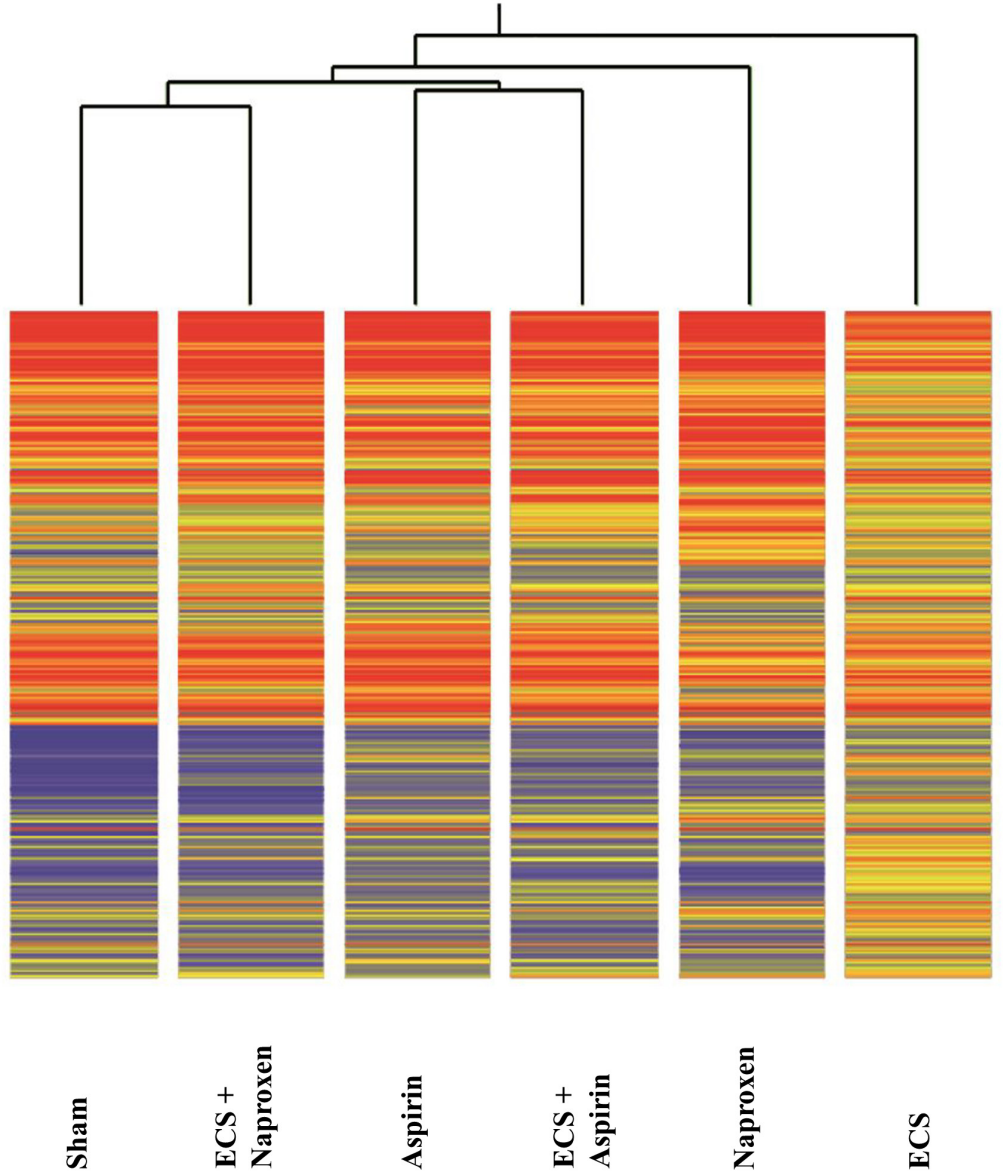
Unsupervised hierarchical cluster analysis (HCA) displaying the overall expression of 1135 miRNAs in the lung of 10-week old A/J mice of both genders, as related to exposure to ECS since birth and/or administration od either aspirin or naproxen after weaning Each line reports the expression intensity of a single miRNA on a color scale (red, high; yellow, intermediate; blue, low).

We performed the analysis of 10 samples pooled from the 5 mice composing each experimental group. The overall miRNA expression profiles of the 1135 mouse miRNAs analyzed was evaluated by supervised hierarchical cluster analysis (HCA, Figure 1). Both NSAIDs modulated the baseline miRNA expression observed in Sham to some extent. At the first level of the hierarchical cluster, the Aspirin profile was located in a dendrogram branch different from Sham but it was still connected with it at the second level of the hierarchical cluster. Naproxen was connected with Sham at the third level of the hierarchical cluster. MCS considerably altered the baseline miRNA expression, its profile being at the opposite side of the dendrogram compared to Sham. Both NSAIDs tended to approach the Sham profiles in ECS-exposed mice. In particular, the ECS + Aspirin profile became connected with the Sham profile at the second level of the hierarchical tree, whereas the ECS + Naproxen profile was quite effective in counteracting ECS-induced alterations, the ECS + naproxen profile being directly connected with Sham at the first level of the hierarchical tree.

**Figure 2:**
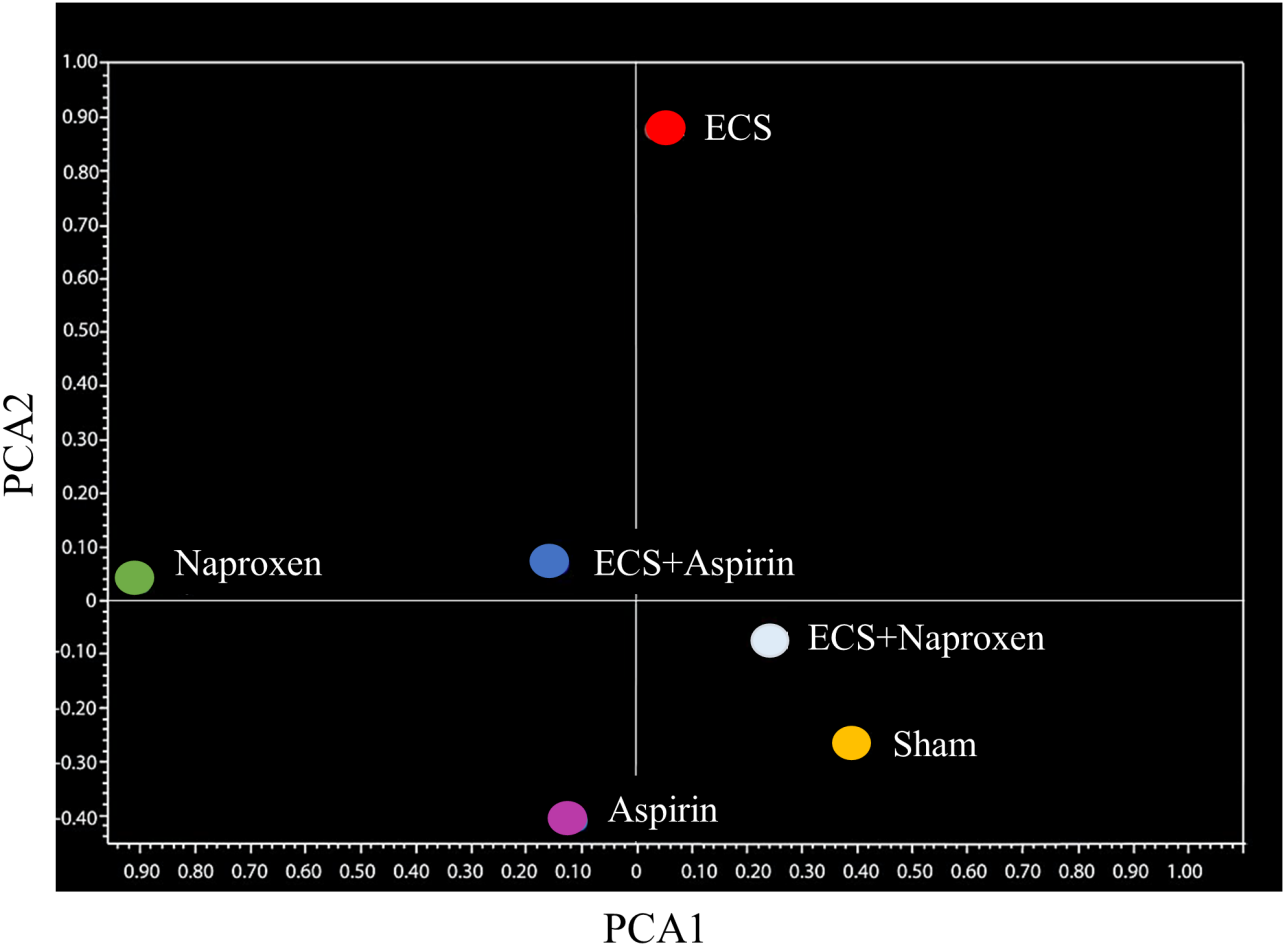
Bidimensional principal component analysis of variance (PCA) showing the overall expression of 1135 miRNAs in the lung of 10-week old A/J mice of both genders, as related to exposure to ECS since birth and/or administration of either aspirin or naproxen after weaning

These conclusions were further supported by unsupervised bidimensional principal component analysis of variance (PCA, Figure 2). The physiological profile of miRNAexpression in the lung of sham-exposed mice was substantially modified in the lung of ECS-exposed mice, as inferred from the finding that the Sham symbol and the ECS symbol were located far away in the graph. Administration of aspirin to smoke-free mice slightly modified the baseline miRNA profiles, as indicated by the finding that the Sham symbol was nearby the Aspirin symbol but in a different quadrant. Conversely, naproxen altered the baseline miRNA expression in a more evident way, with the Sham symbol and the Naproxen symbol falling in two opposite quadrants. Administration of either NSAID to ECS-exposed mice tended to modulate the overall miRNA expression compared to ECS-exposed mice in the absence of chemopreventive agents. This effect was less evident in ECS-exposed mice treated with aspirin, whose symbol (ECS + Aspirin) was half way between the ECS and the Sham symbols. In ECS-exposed mice treated with naproxen, the symbol (ECS + Naproxen) was quite close to the Sham symbol.

**Figure 3:**
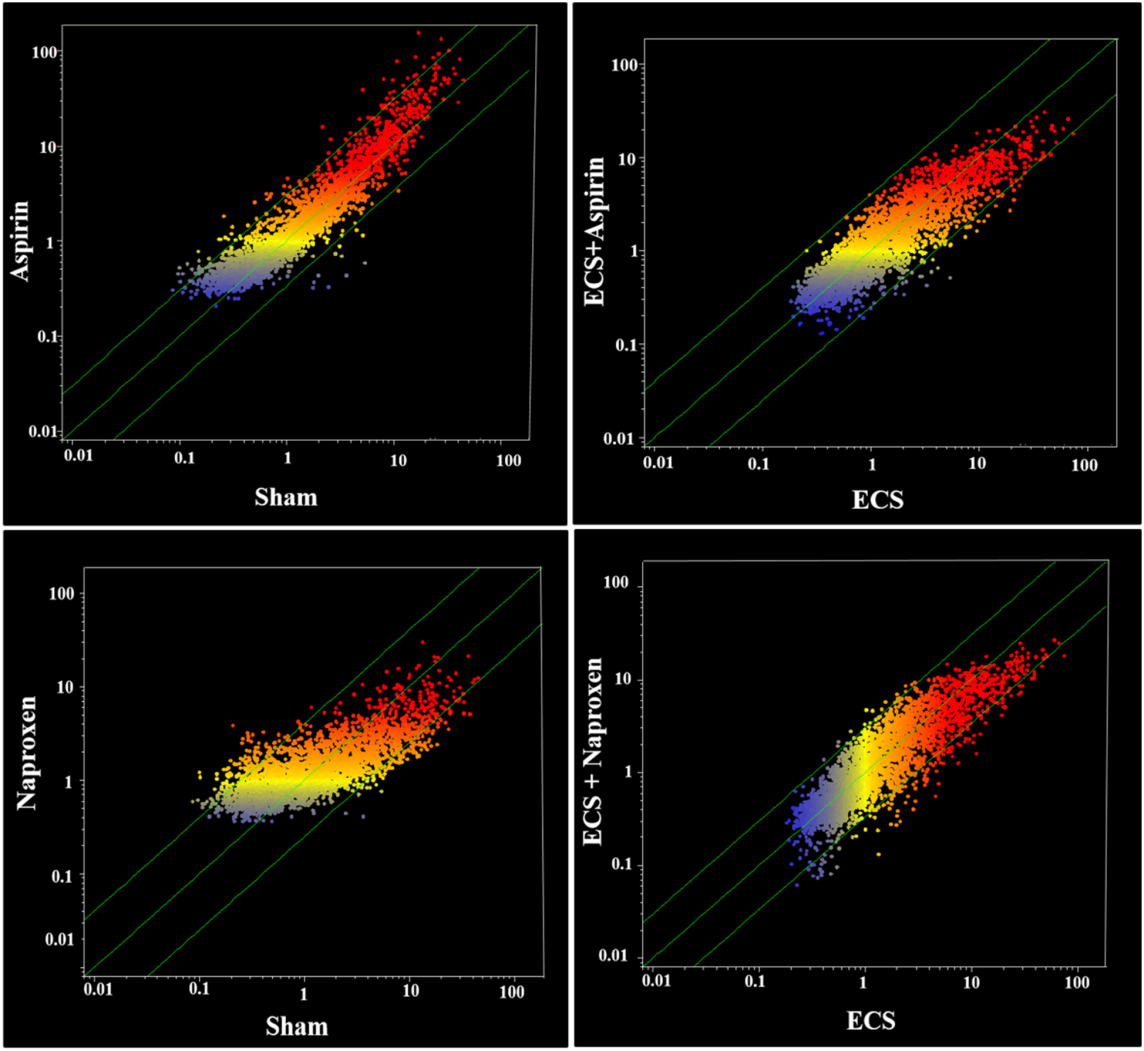
Scatter-plot analyses comparing the overall expression of 1135 miRNAs **(a)** in the lung of sham-exposed (x-axis) *vs.* aspirin-treated (y-axis) 10-week old A/J mice of both genders (upper left panel) and of ECS-exposed (x-axis) *vs*. ECS-exposed and aspirin-treated (y-axis) mice (bottom left panel) and **(b)** in the lung of sham-exposed (x-axis) vs. naproxen-treated (y-axis) 10-week old A/J mice of both genders (upper right panel) and of ECS-exposed (x-axis) vs. ECS-exposed and naproxen-treated (y-axis) mice (bottom right panel). Dots falling outside the diagonal two-fold variation interval (diagonal green lines) correspond to miRNAs dysregulated more than two-fold in their expression by either aspirin or naproxen. Upregulated miRNAs are located in the upper-left area and downregulated miRNAs are in the lower-right area. Each dot represents a miRNA, whose expression intensity can be inferred from the position in the x and y axes, according to a color scale (blue, low; yellow, medium; orange to red, high).

In addition, the effects of the two NSAIDs on miRNA expression were evaluated by scatter-plot analyses. Figure 3 (left panels) shows scatter-plots relative to the effects of aspirin either in sham-exposed mice (upper panel) or in ECS-exposed mice (bottom panel). The comparison of Sham and Aspirin indicates that only a minority of the analyzed miRNAs was located outside the 2-fold variation interval (diagonal green lines). Volcano-plot analyses (not shown), assuming both the 2-fold variation and the *P* < 0.05 statistical significance threshold, indicated that only 7 miRNAs out of a total of the 1135 mouse pulmonary miRNAs tested (0.6%) were modulated by the oral administration of aspirin. Table 1 shows the expression of those miRNAs that were modulated by aspirin either in sham-exposed mice or in MCS-exposed mice. MiRNA functional annotations in Tables 1–8 were generated according to PubMed searches and our previous papers [27, 29–31]. Compared to sham, aspirin dysregulated miRNAs involved in COX-2 regulation, stress response, inflammation, cell proliferation, and apoptosis. Compared to ECS, aspirin dysregulated three miRNAs involved in COX regulation, 2 miRNAs involved in cell proliferation, and 1 miRNA involved in inflammation.

**Table 1:** miRNA expression in the lung of 10-week old A/J mice of both genders, as related to exposure to ECS since birth and/or administration of aspirin after weaning

miRNA	Treatment of mice				Main regulated functions
	Sham	Aspirin	ECS	ECS + Aspirin	
*miR-16*	0.18	1.32^a^	1.00^a^	1.26^a^	Apoptosis
*miR-133*	0.77	0.14^a^	0.97	0.11^a,b^	Inflammation
*miR-137*	0.49	1.20^a^	0.77^a^	1.65^a,b^	Cell proliferation, COX-2 negative regulator
*miR-191*	0.89	1.08	0.43^a^	1.04^b^	COX regulation, Cell proliferation
*miR-199b*	0.94	1.04	1.72	0.86^b^	COX activation
*miR-223*	1.08	1.05	0.51^a^	1.02	Stress response, Protein repair, k-*Ras* regulation
*miR-543*	1.17	0.27^a^	1.85	1.14	Stress response, Inflammation

The results are expressed in FU (Fluorescence Units).

Statistical analysis: ^a^*P* < 0.05 compared with Sham; ^b^*P* < 0.05 compared with ECS-exposed mice in the absence of chemopreventive agents.

**Table 2:** miRNA expression in the lung of 10-week old A/J mice of both genders, as related to exposure to ECS since birth and/or administration of naproxen after weaning

miRNA	Treatment of mice				Main regulated functions
	Sham	Naproxen	ECS	ECS + Naproxen	
*miR-16*	0.18	3.52^b^	1.00	1.00	Apoptosis
*miR-27a	0.71	0.37^a^	1.40	0.56^c^	Cell proliferation, Stress response, Protein repair
*miR-101*	0.67	2.09^a^	1.06	0.61	COX activation
*miR-133*	0.77	0.32^a^	0.97	0.46	Inflammation
*miR-137*	0.49	2.26^a^	0.77	1.43	Cell proliferation, COX-2 negative regulator
*miR-146a*	0.98	3.18^a^	0.46	0.87	COX regulation, Regulation of NFκB stress response, Inflammation
*miR-181c	1.02	0.54^a^	1.91	0.86	NFκB stress response
*miR-191	0.89	1.94^a^	0.43^b^	0.85	COX activation, Cell proliferation
*miR-199b*	0.94	3.11^b^	1.72	1.09	Inflammation, Regulation of mTOR activation
*miR-203*	0.85	1.62^b^	0.48	0.73	Cell proliferation, Angiogenesis
*miR-218*	1.44	4.58^a^	0.66	1.21	Stress response, Oncogene k*Ras* regulation, Antioxidant
*miR-223	1.08	4.17^b^	0.51^c^	1.09	-
*miR-375*	1.07	2.20^b^	0.63	1.16	Inflammation (Prostaglandin activation), Cell differentiation
*miR-409	0.85	1.67^a^	0.42	1.54^d^	-
*miR-499*	0.88	1.93^b^	0.43	1.15	Cell proliferation, Cell differentiation
*miR-509	1.08	2.43^a^	0.46	2.56^d^	-
*miR-532	1.11	2.36^b^	0.52^b^	2.33^c^	Gene transcription, apoptosis
*miR-543*	1.17	0.98	1.85	0.78^c^	Stress response, inflammation
*miR-708	1.95	0.88^a^	2.85	1.98	Stress response, NFkB activation

The results are expressed in FU (Fluorescence Units).

Statistical analysis: ^a^*P* < 0.05 and ^b^*P* < 0.01, as compared with Sham; ^c^*P* < 0.05 and ^d^*P* < 0.01 as compared with ECS-exposed mice in the absence of chemopreventive agent.

*Altered by ECS and restored by naproxen at least 2-fold.

**Table 3:** Differentially expressed miRNAs in the lung of female *vs*. male mice evaluated by volcano-plot analysis, as related to exposure of 10-week old A/J mice to ECS since birth and/or treatment with NSAIDs after weaning

miRNA	Treatment of mice						Main regulated functions
	Sham	Naproxen	Aspirin	ECS	ECS + Naproxen	ECS + Aspirin	
*let-7e-5p*	7.14/2.62	6.10/2.21	3.13/3.74	3.41/5.23	12.04/2.80	2.11/3.42	Cell proliferation
*miR-1*	8.74/6.93	4.80/0.51	4.93/3.03	5.44/0.80	7.32/5.54	3.74/3.86	Apoptosis; Cardiogenesis; Myogenesis
*miR-7b-3p*	1.81/2.37	3.32/2.62	3.88/1.77	2.60/0.79	2.14/2.71	1.97/1.67	Inflammation (*NFATc1* and *c-Fos* regulation)
*miR-23c*	2.65/6.08	3.21/0.71	8.35/9.32	4.45/0.87	1.80/13.83	7.71/2.56	Gene transcription
*miR-30b-5p*	2.93/7.18	9.75/1.93	10.58/5.10	7.69/0.88	1.83/4.34	4.43/2.63	Intercellular adhesion; Protein repair; NFkB activation; Cell cycle; EGF activation; Stem cell recruitment; Multidrug resistance; Mammary gland development
*miR-32-3p*	11.07/6.19	16.93/5.76	11.36/5.39	9.08/5.75	14.52/3.48	3.22/4.31	Apoptosis; Oligodendrocyte function and development
*miR-92a*	10.52/3.37	2.33/0.99	1.68/2.34	4.02/6.72	13.43/4.60	4.32/3.73	DICER regulation
*miR-125b*	4.76/2.20	3.88/1.11	3.54/4.47	2.92/1.96	3.57/2.50	3.46/2.71	Oncogene *ERBB* regulation; Vitamin D receptor; Inflammation
*miR-181b-5p*	2.08/2.68	1.59/13.69	1.84/2.24	1.36/5.47	1.57/5.39	5.44/1.53	NFkB stress response; Cell proliferation, k-R*as* suppression
*miR-200c*	4.65/3.95	0.38/1.85	2.22/3.34	4.22/0.69	3.07/5.86	3.25/2.64	Apoptosis; Intracellular trafficking; Protein repair
*miR-204-3p*	11.06/3.58	8.19/1.38	5.23/7.74	11.13/13.78	14.65/3.59	5.96/5.72	Epithelial mesenchymal transition; Apoptosis; Cell proliferation; Oncogene (*VHL*) suppression; Removal of cancer cell by mitochondrial driver autophagy
*miR-206*	2.72/4.84	4.57/0.58	13.90/3.87	4.98/0.74	2.26/6.57	4.57/3.12	Myoblast differentiation and proliferation
*miR-218*	0.74/0.79	0.88/3.06	1.10/0.73	0.76/1.93	0.67/1.03	1.04/0.85	Stress response; Oncogene k*-Ras* activation; Antioxidant; Cell proliferation
*miR-297b*	0.64/0.92	0.47/3.63	0.55/0.48	0.76/0.47	1.32/0.45	0.60/0.71	Protein repair; Cell proliferation
*miR-298-3p*	1.20/1.89	1.47/0.81	3.06/1.46	1.94/0.36	0.83/2.20	1.87/1.41	Doxorubicin chemoresistance; Expression of mouse β-amyloid precursor protein-converting enzyme 1
*miR-344d-3-5p*	0.79/0.72	1.02/0.79	1.03/1.01	0.76/3.37	0.68/1.03	1.00/0.70	Synapse development and plasticity
*miR-350-5p*	0.22/0.35	0.33/3.59	0.47/0.47	0.30/0.29	0.12/0.53	0.76/0.28	Inflammation (Increase in unphosphorylated NFATc3 and its nuclear translocation)
*miR-421-3p*	1.65/2.83	6.67/1.72	7.57/2.35	2.86/0.30	2.13/1.57	1.24/2.25	Post-transcriptional regulation of ACE2
*miR-466a-5p*	4.36/3.17	0.53/5.42	2.96/1.55	3.63/1.55	5.95/0.94	0.91/2.63	Cell proliferation; k*-Ras* activation; Apoptosis; Protein repair; Cell proliferation
*miR-467h*	6.37/3.83	5.70/0.52	3.94/2.10	4.44/1.83	9.04/2.07	1.80/1.94	Cell proliferation; Protein synthesis
*miR-483-5p*	3.00/1.94	0.89/2.17	2.26/2.27	3.79/1.37	2.52/0.88	1.34/5.12	Epithelial-mesenchymal transition; Prometastatic fuction (RhoGDI1 and ALCAM regulation); Angiogenesis-inhibiting factor
*miR-519e-5p*	2.58/0.98	1.92/0.85	0.99/1.56	2.45/6.68	4.89/0.63	1.04/2.48	Cell proliferation; Oncogene (*HuR*) suppression; Associated with the expression of IGF2 IGF1, INHBA, ADIPOR2, INHBE, STC2, ADCYAP1, PRRG2, ADM2, DNASE2
*miR-550b-5p*	3.04/1.77	2.03/0.81	1.51/1.46	2.11/7.37	6.31/1.08	1.12/3.77	-
*miR-627*	7.78/2.66	12.97/0.86	10.83/9.70	10.51/3.98	10.77/1.45	4.53/10.23	Mediates tumor-suppressive epigenetic activities of vitamin D
*miR-632*	5.15/5.80	2.50/1.45	6.04/5.06	4.63/4.77	3.34/13.63	5.92/5.30	Downregulation of DNAJB6 (HSP40 family); Implicated in invasive activity of breast cancer cells
*miR-664b-5p*	5.26/1.82	1.24/0.53	1.36/1.99	3.49/3.83	7.41/1.79	2.52/4.02	Decreases MAPK-1 expression which reduces TNF-α
*miR-708-3p*	0.74/0.41	0.81/4.93	0.47/0.58	0.69/2.53	0.60/0.06	0.17/0.48	Stress response; NFkB activation; Cell proliferation; Apoptosis
*miR-709*	23.81/11.36	25.91/1.34	13.01/13.18	17.28/9.55	34.04/7.36	6.73/12.81	Stress response, Inflammation, Lysosome activation
*miR-711*	14.13/6.57	14.58/1.79	5.95/12.72	9.09/14.05	12.79/3.18	7.82/12.19	Inhibition of heart fibrosis after heart infarction
*miR-765*	32.42/12.43	23.93/2.74	16.89/14.49	27.22/9.88	30.79/6.87	7.05/11.52	Associated with the expression of IGF2, GALP, TTR, TOR2A, STC2, CSH2, ADIPOR2 OXT

The reported values indicate the intensity of miRNA expression (Fluorescence Units) in females and males (females/males).

**Table 4:** miRNA levels (Fluorescence Units), as inferred from volcano-plot analyses, in the blood serum of 10-week old A/J mice of both genders, as related to exposure to ECS since birth and/or administration of aspirin after weaning

miRNA	Sham	Naproxen	Aspirin	ECS	ECS + Naproxen	ECS + Aspirin	Main regulated functions
*miR-1a*	2.45	3.54	2.91	6.17	5.26	4.20	Urethane exposure
*miR-31-3p*	0.23	0.32	0.31	0.57	0.37	0.53	Cell proliferation
*miR-92a*	10.49	21.99	12.98	51.12	8.61	23.06	Cell proliferation and invasion
*miR-96-5p*	0.24	0.45	0.42	0.24	1.92	0.37	Adhesion and MAPK signaling; Regulation of tumor suppressor *PTEN*; Cell proliferation (targeting *FOXO1*, *FOXO3a*, p27Kip1, and p21Cip1)
*miR-130a*	0.35	0.43	0.52	1.59	0.54	1.82	Regulates autophagy of endothelial progenitors cells
*miR-140-5p*	0.63	0.73	0.92	0.61	1.29	0.82	Involved in cartilage homeostasis and osteoarthritis
*miR-148b-5p*	0.61	2.88	0.59	0.58	0.65	0.53	Apoptosis; Tumor suppressor in hepatocellular carcinoma
*miR-183-5p*	4.30	8.97	8.02	9.81	6.42	5.04	Adhesion and MAPK signaling; Regulation of the tumor suppressor *PTEN*; Cell proliferation (targeting *FOXO3a*, p27Kip1, and p21Cip1
*miR-186-3p*	1.15	1.73	1.98	2.61	1.23	2.02	Modulates PTTG1 protein expression (function in NSCLC invasion/metastasis)
*miR-193b-5p*	3.49	5.45	6.17	7.35	4.52	4.37	Repression of myogenic markers Pax3 and MyoD; Upregulation of adipogenesis markers (AdipoQ, PPARγ, Cebpα and Fabp4) and brown fat markers (Ucp1, Cidea, Prdm16 and PPARα)
*miR-201-3p*	0.84	2.86	3.61	0.83	1.12	0.86	-
*miR-204*	13.31	35.48	17.75	80.61	47.81	62.34	Epithelial mesenchymal transition; Apoptosis; Cell proliferation; Oncogene (*VHL*) suppression; Removal of cancer cell by mitochondrial driver autophagy
*miR-218-5p*	0.18	0.49	0.24	0.97	0.48	0.67	Differentiation of bone marrow stromal cells by activating a positive Wnt signaling loop; Endothelial cell migration during blood vessel development
*miR-291a-3p*	0.37	0.27	0.44	0.38	0.56	0.38	Differentiation of embryonic stem cells
*miR-297a*	0.21	0.30	0.34	0.77	0.49	0.56	Cell proliferation
*miR-301a*	3.34	3.66	3.65	14.85	8.38	8.45	Targets *PTEN* and activates the Wnt/-catenin pathway
*miR-324-3p*	1.25	0.76	1.18	1.26	1.77	1.52	Induces promoter-mediated expression of the *RelA* gene
*miR-331-5p*	1.13	1.22	1.72	2.35	2.82	2.02	Inhibits expression of neuropilin-2, a receptor implicated in neuronal development
*miR-340*	2.66	4.67	4.34	11.61	9.14	8.85	Dendrite formation and melanosome transport; Inhibits cell proliferation and migration in osteosarcoma
*miR-344c-5p*	0.56	1.67	1.35	0.56	0.81	0.67	Neural development; Influences the expression of genes involved in one-carbon metabolism (DNA synthesis and methylation, amino acid metabolism) and cell proliferation
*miR-363-5p*	13.19	18.8	16.84	13.36	5.37	7.58	Cardiac cell differentiation; Expression of endothelial cell-specific genes
*miR-376b*	0.15	0.30	0.27	0.64	0.49	0.61	Angiogenesis
*miR-377-5p*	1.22	1.48	1.73	2.61	2.36	2.81	Regulates heme oxygenase-1 protein expression
*miR-380*	2.81	2.93	3.01	6.22	3.23	3.44	Represses p53 to control cell survival
*miR-411-5p*	2.17	3.35	3.32	2.12	1.46	1.57	Neuronal growth
*miR-450b*	0.10	0.45	0.16	0.53	0.61	0.46	-
*miR-455-5p*	0.48	2.83	0.41	0.47	0.37	0.62	TGF signaling; Enhances cancer growth and metastasis *in vivo*
*miR-466i*	0.82	1.74	1.28	2.05	2.21	1.02	Involved in fattyacid metabolism
*miR-487b*	1.23	1.40	1.27	6.14	4.91	4.14	Promotes endothelial cell proliferation, migration, and invasion
*miR-495*	0.29	0.39	0.35	1.26	0.78	1.18	Inhibits the migration and invasion of human gastric cancer cells
*miR-500-3p*	1.31	3.04	3.67	1.33	2.84	1.48	Activator of the NF-ĸB signaling pathway
*miR-551b*	2.56	3.60	3.07	13.03	8.98	9.89	Involved in chemoresistance and apoptosis resistance in lung cancer cells
*miR-667-5p*	2.51	6.29	4.26	2.42	2.41	2.96	-
*miR-709*	16.02	32.39	28.65	38.44	22.44	19.08	Cell differentiation; Counteracts aberrant DNA hypomethylation
*miR-763*	8.15	9.39	9.07	14.45	5.23	6.84	-
*miR-764*	0.13	0.18	0.17	0.58	0.41	0.54	Cell differentiation
*miR-874-3p*	24.98	32.6	31.24	43.17	18.38	17.88	Neuronal survival and apoptosis

**Table 5:** miRNA expression either in lung or blood serum of 7.5-month old Swiss mice of both genders exposed to MCS during the first 4 months of life and treated with aspirin after weaning, as related to microadenoma multiplicity

miRNA	Lung	Blood	Main regulated functions
*miR-16*		0.08	Apoptosis
*miR-30c*	0.2	0.13	Intercellular adhesion, protein repair, NFkB activation, cell cycle, EGF activation, stem cell recruitment, multidrug resistance, mammary gland development
*miR-133*		0.1	Inflammation
*mmu- miR-137*		0.49	Cell proliferation, COX-2 negative regulator
*miR-181b*	0.13	0.24	NFκB stress response
*miR-183*	0.29	0.20	Adhesion and MAPK signaling, regulation of the tumor suppressor *PTEN*, cell proliferation (targeting *FOXO3a*, p27Kip1, and p21Cip1
*miR-191*		0.35	COX regulation, cell proliferation
*miR-301a*	0.14	0.16	Targets *PTEN* and activates the Wnt/b-catenin pathway
*miR-350*	0.1	0.19	Inflammation (increase in unphosphorylated NFATc3 and its nuclear translocation)
*miR-466a-3p*	0.07	0.40	Cell proliferation, k*-Ras* activation, apoptosis, protein repair, cell proliferation
*miR-466i-3p*	0.16	0.35	Involved in fatty acid metabolism
*miR-500*	0.1	0.1	Activator of the NF-ĸB signaling pathway
*miR-709*	0.1	0.13	Stress response, inflammation, lysosome activation

The numbers indicate the ratio of miRNA expression (fold-variation) between mice bearing <10 vs. >10 microadenomas.

**Table 6:** miRNA expression either in lung or blood serum of 7.5-month old Swiss mice of both genders exposed to MCS during the first 4 months of life and treated with aspirin after weaning, as related to the presence of lung adenomas

miRNA	Lung	Blood	Main regulated functions
*miR-30e*	0. 08		Intercellular adhesion, protein repair, NFkB activation, cell cycle, EGF activation, stem cell recruitment, multidrug resistance
*miR-32*	0.44		Apoptosis
*miR-181b*	0.35		NFkB stress response, cell proliferation, k-R*as* suppression
*miR-183*		1.74	Adhesion and MAPK signaling, regulation of the tumor suppressor *PTEN*, cell proliferation (targeting *FOXO3a*, p27Kip1, and p21Cip1)
*miR-301a*	0.36		Targets *PTEN* and activates the Wnt/b-catenin pathway
*miR-350*	0.39		Inflammation (increase in unphosphorylated NFATc3 and its nuclear translocation)
*miR-380*	0.14		Represses p53 to control cell survival
*miR-466a*	0.29		Cell proliferation, k*-Ras* activation, apoptosis, protein repair, cell proliferation
*miR-466i*	0.39		Involved in fatty acid metabolism
*miR-543*	0.15		Stress response, inflammation

The numbers indicate the ratio of miRNA expression (fold-variation) between adenoma-bearing vs adenoma-free mice.

**Table 7:** miRNA expression either in lung or blood serum of 7.5-month old Swiss mice of both genders exposed to MCS during the first 4 months of life and treated with naproxen after weaning, as related to microadenoma multiplicity

miRNA	Lung	Blood	Main regulated functions
*miR-16*	4.20		Apoptosis
*miR-27a*	4.59		Cell proliferation, stress response, protein repair
*miR-30*	0.19		Intercellular adhesion, protein repair, NFkB activation, cell cycle, EGF activation, stem cell recruitment, multidrug resistance, mammary gland development
*miR-92a*	6.35		DICER regulation
*miR-101*	4.56		COX activation
*miR-133a*	3.26		Inflammation
*miR-137*	4.88		Cell proliferation, COX-2 negative regulator
*miR-181b*	3.32	1.30	NFκB stress response
*miR-148b*	4.03		Apoptosis, tumor suppressor in hepatocellular carcinoma
*miR-186*	6.06		Modulates PTTG1 protein expression (function in NSCLC invasion/metastasis)
*miR-203b*	5.55		Cell proliferation, angiogenesis
*miR-301a*	0.27		Stress response, oncogene activation
*miR-324*		1.32	Induces promoter-mediated expression of the *RelA* gene
*miR-331*	3.23		Inhibits expression of neuropilin-2, a receptor implicated in neuronal development
*miR-344b*	3.25		Cell plasticity, influences the expression of genes involved in one-carbon metabolism (DNA synthesis and methylation, amino acid metabolism) and cell proliferation
*miR-344d*		1.49	Cell plasticity, influences the expression of genes involved in one-carbon metabolism (DNA synthesis and methylation, amino acid metabolism) and cell proliferation
*miR-344e*	6.15		Influences the expression of genes involved in one-carbon metabolism (DNA synthesis and methylation, amino acid metabolism) and cell proliferation
*miR-344f*	3.18	1.33	Neural development, influences the expression of genes involved in one-carbon metabolism (DNA synthesis and methylation, amino acid metabolism) and cell proliferation
*miR-363*	6.40		Cardiac cell differentiation, expression of endothelial cell-specific genes
*miR-375*	3.75		Inflammation (prostaglandin activation), Cell differentiation
*miR-380*	4.27		Represses p53 to control cell survival
*miR-409b*	3.22		-
*miR-455*	3.10		TGF signaling, enhances cancer growth and metastasis *in vivo*
*miR-466a*		1.42	Involved in fatty acid metabolism
*miR-487b*			Promotes endothelial cell proliferation, migration, and invasion
*miR-499a*			Cell proliferation, cell differentiation
*miR-532*	3.85		Gene transcription, apoptosis
*miR-543*			Stress response, inflammation
*miR-551b*		1.47	DNA repair, inflammation, cell proliferation
*miR-664b*			Decreases MAPK-1 expression which reduces TNF-α
*miR-708*	3.04	0.62	Stress response, NFkB activation

The numbers indicate the ratio of miRNA expression (fold-variation) between mice bearing <10 vs. >10 microadenomas.

**Table 8:** miRNA expression either in lung or blood serum of 7.5-month old Swiss mice of both genders exposed to MCS during the first 4 months of life and treated with naproxen after weaning, as related to the presence of lung adenomas

miRNA	Lung	Blood	Main regulated functions
*miR-30b*		0.27	Intercellular adhesion, protein repair, NFkB activation, cell cycle, EGF activation, stem cell recruitment, multidrug resistance
*miR-30c*	0.47		Intercellular adhesion, protein repair, NFkB activation, cell cycle, EGF activation, stem cell recruitment, multidrug resistance
*miR-101b*		1.86	COX activation
*miR-125b*		0.31	Oncogene *ERBB* regulation, vitamin D receptor, inflammation
*miR-206*		0.27	Myoblast differentiation and proliferation
*miR-291a*		0.47	Differentiation of embryonic stem cells
*miR-344b*	4.83		Cell plasticity
*miR-344h*	2.05		Cell plasticity
*miR-483*	5.45		Epithelial-mesenchymal transition, prometastatic fuction (RhoGDI1 and ALCAM regulation), angiogenesis-inhibiting factor
*miR-711*	0.1	0.52	Tissue fibrosis

The numbers indicate the ratio of miRNA expression (fold-variation) between adenoma-bearing vs adenoma-free mice.

Figure 3 (right panels) shows scatter-plots relative to the effects of naproxen either in sham-exposed mice (upper panel) or in ECS-exposed mice (bottom panel). Compared to aspirin, a higher number of the analyzed miRNAs was located outside the 2-fold variation interval (diagonal green lines). Volcano-plot analyses (not shown), assuming both the 2-fold variation and the *P* < 0.05 statistical significance threshold, indicated that 19 miRNAs out of a total of 1135 mouse pulmonary miRNAs tested (1.5%) were modulated by the oral administration of naproxen. By comparing ECS with ECS + naproxen (bottom right panel of Figure 3), a number of miRNAs were located outside the 2-fold variation interval (diagonal green lines), indicating an effect of naproxen in counteracting ECS-induced miRNA alterations. Volcano-plot analyses (not shown), indicated that 12 miRNAs out of a total of 53 ECS-dysregulated miRNAs (22.6%) were modified in their expression by the oral administration of naproxen. Table 2 showsthe expression ofthose miRNAs that were modulated by naproxen either in sham-exposed mice or in ECS-exposed mice. The functions modulated by naproxen in sham-exposed mice covered a variety of biological processes, including COX regulation (4 miRNAs), prostaglandin activation (1), stress response (3), antioxidant activity (1), protein repair (1), inflammation (3), cell proliferation (5), cell differentiation (2), apoptosis (2), angiogenesis (1), gene transcription (1), and regulation of NFκB (3), mTOR (1) and k*Ras* (1). A significant dysregulation by naproxen in ECS-exposed mice was observed for a more limited number of miRNAs involved in stress response (2 miRNAs), protein repair (1), inflammation (1), cell proliferation (1), and apoptosis (1).

The interindividual variability of microarray results was evaluated in ECS-exposed mice treated with aspirin, which were analyzed individually for miRNA expression. The mean expression intensity of the 1135 mouse miRNA tested in these 5 mice was 3.3 ± 0.27 FU (mean ± SE). Accordingly, these results indicate that the SE accounted for the 8.2% of the mean.

#### Intergender differences in pulmonary miRNA expression

**Figure 4:**
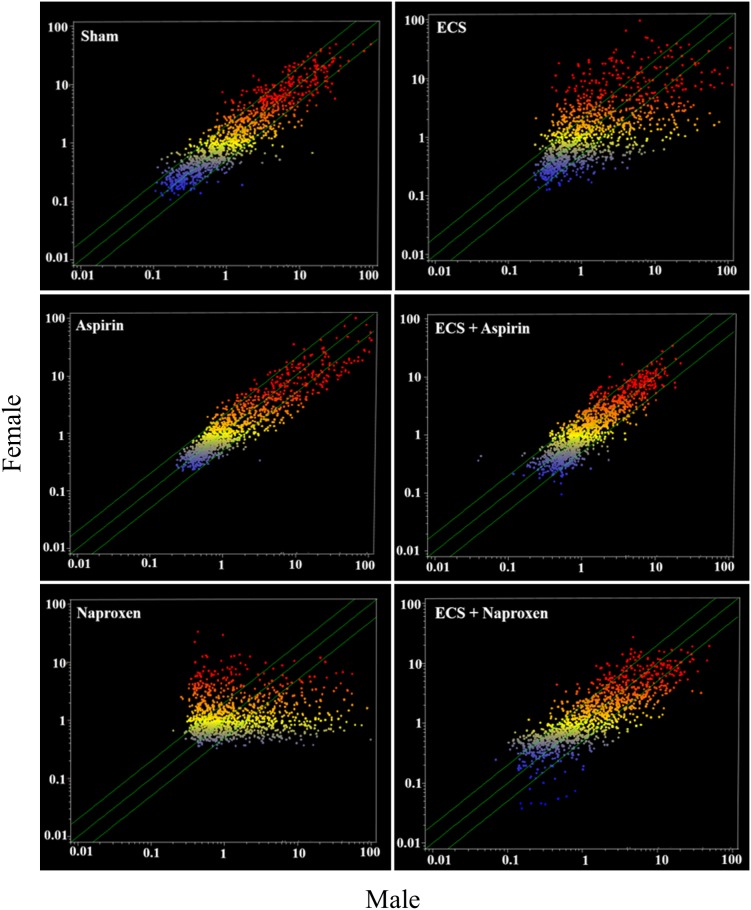
Scatter-plots comparing the overall expression of 1135 miRNAs in the lung of male and female 10-week old A/J mice, as related to exposure to ECS and/or treatment with aspirin or naproxen Each dot represents a miRNA, whose expression intensity can be inferred from the position in the x- and y-axes, according to a color scale (blue, low; yellow, medium; orange to red, high). The diagonal belts indicate the 2-fold variation interval. Symbols falling in the upper left area denote a higher expression of miRNAs in females compared to males, whereas symbols falling in the lower right area denote a higher expression of miRNAs in males compared to females.

Figure 4 shows scatter-plots comparing male and female mice belonging to the 6 experimental groups (Sham, Aspirin, Naproxen, ECS, ECS + Aspirin, and ECS + Naproxen). Many miRNAs were differentially expressed in males and females in all experimental groups. The most striking differences occurred in ECS-exposed mice and in naproxen-treated mice, in which the expression of a number of miRNAs was higher either in males (dots falling outside the diagonal belts in the lower right portion of the graph) or in females (dots falling outside the diagonal belts in the upper left portion of the graph). Minor differences were observed in Sham, Aspirin, and ECS + Aspirin, and intermediate differences were observed in ECS + Naproxen. The identity of miRNAs undergoing differential intergender expression is reported in Table 3. Assuming a >2-fold difference in miRNA expression as biologically significant, 10 miRNAs were more intensely expressed in females compared to males in sham-exposed mice, whereas only 2 miRNAs were more intensely expressed in males compared to females. In naproxen-treated mice, 18 miRNAs were more intensely expressed in females compared to males, whereas 8 miRNAs were more intensely expressed in males compared to females. In aspirin-treated mice, 6 miRNAs were more intensely expressed in females compared to males, whereas only 1 miRNA was more intensely expressed in males compared to females. In ECS-exposed mice, 13 miRNAs were more intensely expressed in females compared to males, whereas 6 miRNAs were more intensely expressed in males compared to females. In ECS-exposed mice treated with naproxen, 16 miRNAs were more intensely expressed in females compared to males, whereas 7 miRNAs were more intensely expressed in males compared to females. In ECS-exposed mice treated with aspirin, 3 miRNAs were more intensely expressed in females compared to males, whereas 7 miRNAs were more intensely expressed in males compared to females.

As shown in Table 3, important intergender differences were observed for miRNAs playing a role in lung carcinogenesis. In particular, *let-7e*, involved in EGF and k*Ras* modulation, was strikingly downregulated by ECS in females but not in males. Naproxen efficiently counteracted ECS-induced downregulation of this miRNA in females but not in males. *mir-125*, involved in *Erbb2* suppression, was downregulated by ECS in females but not in males. *mir-466a*, activating k*Ras* expression, was downregulated by ECS more strikingly in males than females, and naproxen was effective in counteracting ECS-related downregulation in females but not in males.

#### Validation by qPCR of pulmonary miRNA microarray data

**Figure 5:**
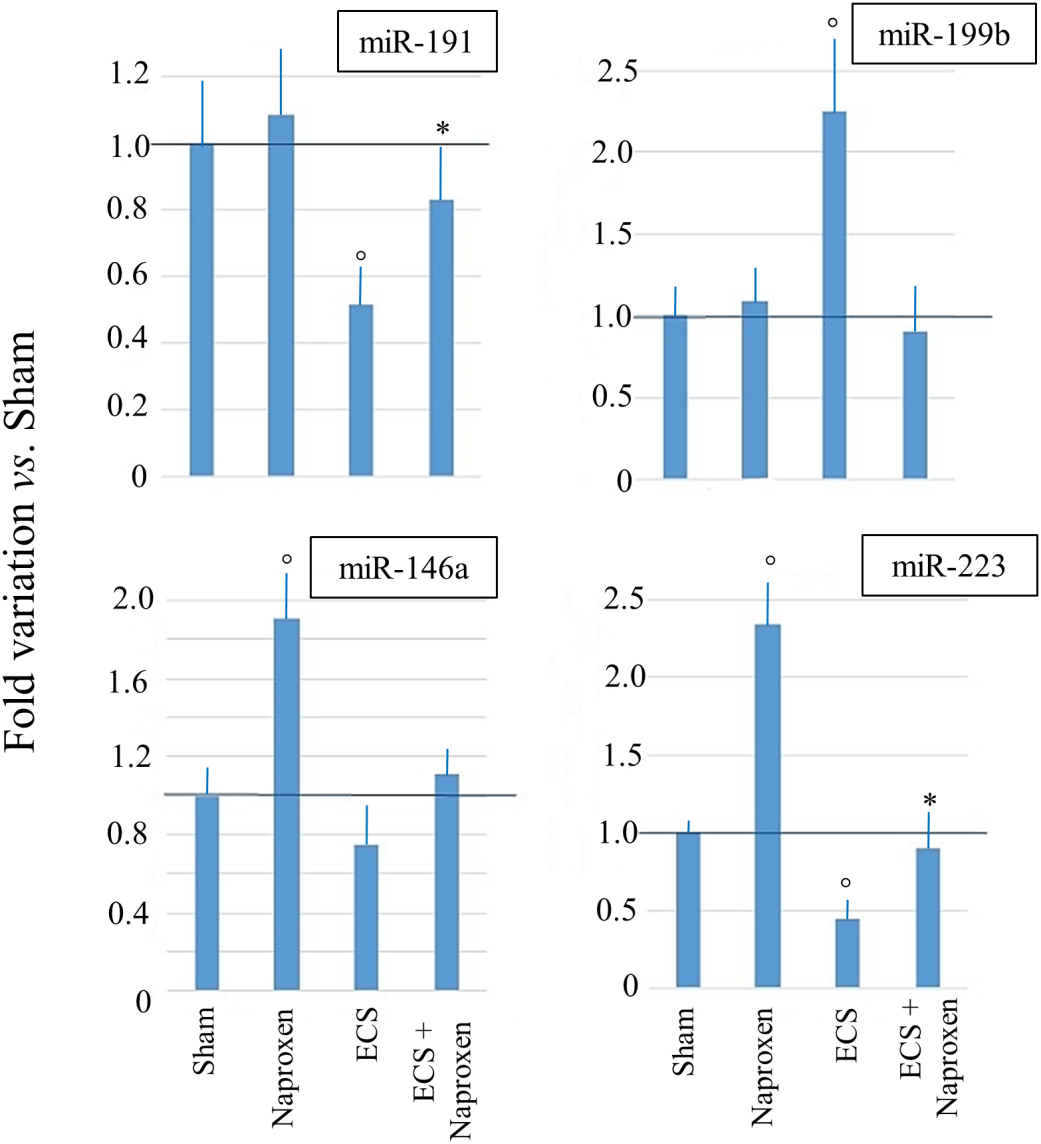
Results of qPCR analyses evaluating the expression of 4 miRNAs in the lung of 10-week old A/J mice of both genders, as related to exposure to ECS since birth and/or administration of either aspirin or naproxen after weaning The data are means + SD of 3 replicates. °*P* < 0.05 *vs.* Sham; *P < 0.05 *vs.* ECS.

The results of qPCR analyses carried out by testing the RNA samples of mice from 6 experimental groups (Sham, Aspirin, Naproxen, ECS, ECS + Aspirin, and ECS + Naproxen) for *miR*-*146a*, *miR-191*, *miR-199b*, and *miR-223* are shown in Figure 5. Although the differences among groups were more attenuated, qPCR analyses confirmed the microarray data.

#### Microarray analysis of blood serum miRNAs

The purified miRNA samples were fully adequate to obtain satisfactory microarray results. Indeed, the spot response rate, i.e. the number of spots providing fluorescent signals, was >90%. The amounts of miRNA extracted from each experimental group ranged between 1.5 and 2.8 μg.

**Figure 6:**
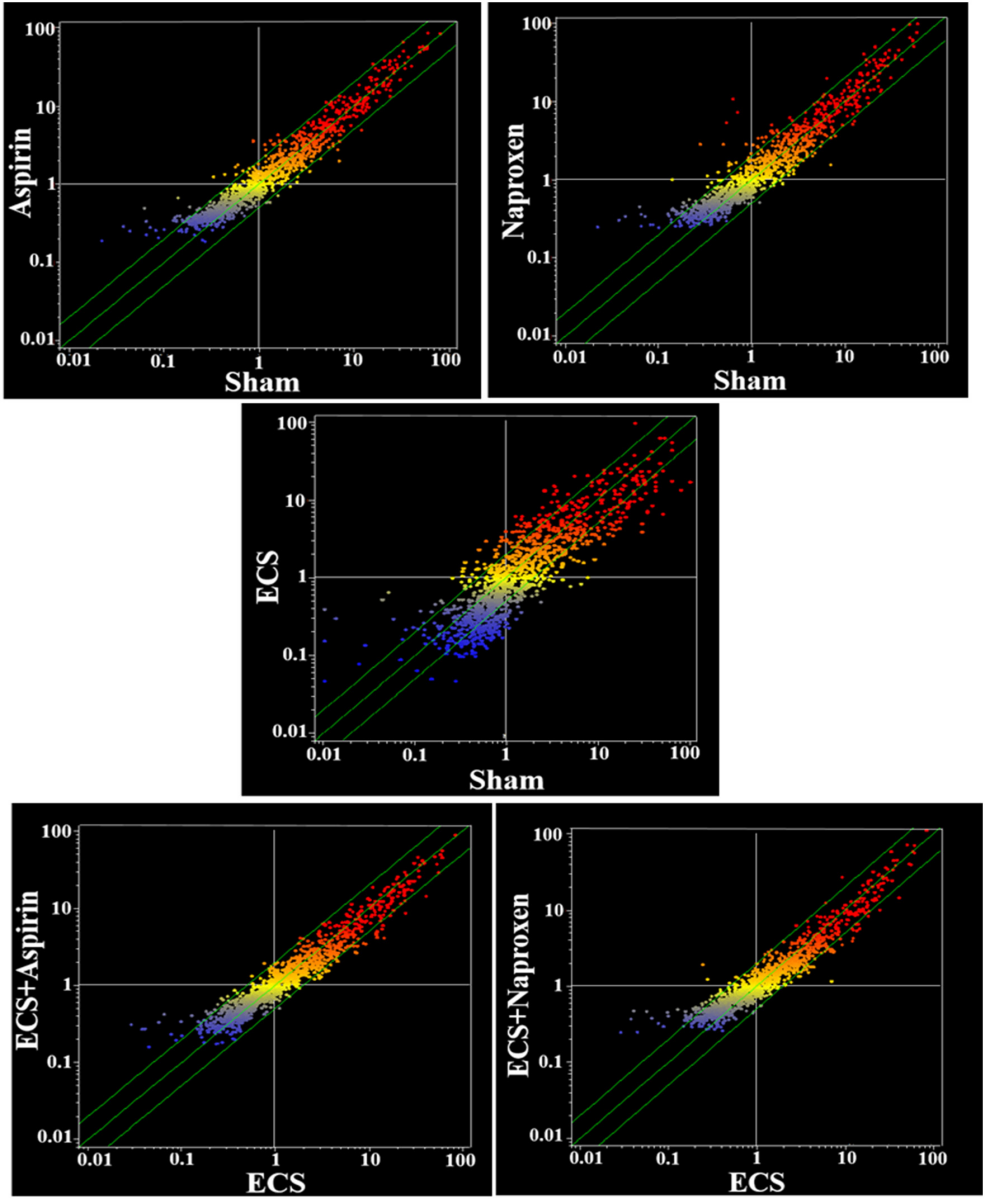
Scatter-plots comparing the overall expression of 1135 miRNAs in the blood serum of 10-week old A/J mice, as related to exposure to ECS since birth and/or treatment with aspirin or naproxen after weaning Each dot represents a miRNA, whose expression intensity can be inferred from the position in the x- and y-axes, according to a color scale (blue, low; yellow, medium; orange to red, high). The diagonal belts indicate the 2-fold variation interval. Symbols falling in the upper left area of each panel denote a higher expression of miRNAs in females compared to males, whereas symbols falling in the lower right area denote a higher expression of miRNAs in males compared to females.

The overall differences in serum miRNA profiles as related to treatment with either aspirin or naproxen in smoke-free mice (Sham) and ECS-exposed mice are shown in the scatter-plots reported in Figure 6. As inferred from the number of miRNAs located outside the two-fold variation interval (green diagonal lines), these differences are more attenuated than those detectable in the lung (see Figure 3 for a comparison). At a glance, the differences appear to be negligible between Sham and Aspirin and between ECS and ECS + Aspirin. They appear to be more pronounced between Sham and Naproxen and between ECS and ECS + Naproxen. In particular, volcano-plot analyses (not shown) identified the numbers of miRNAs that were significantly (>2-fold variation and *P* < 0.05) altered in blood serum as follows: ECS *vs.* Sham = 24; Aspirin *vs*. Sham = 3; Naproxen *vs.* Sham = 13; ECS + Aspirin *vs*. ECS = 5; ECS + Naproxen *vs*. ECS = 9. The identity of these miRNAs is reported in Table 4. Volcano plot analyses (not shown) indicated that no miRNA was differentially expressed in the blood of males and females within the 5 experimental groups.

### Late effects of aspirin and naproxen on miRNA profiles in the lung and blood of Swiss H mice, either unexposed or exposed to MCS

#### Histopathological analyses of the lungs

As we utilized a small-size subset of mice from a broader cancer chemoprevention study [21], the following results were not intended to be used for assessing the efficacy of NSAIDs in preventing MCS-induced lung tumors. In any case, it is noteworthy that no adenoma was detected in sham-exposed mice and that the incidence of adenomas was higher in MCS-exposed mice in the absence of chemopreventive agents (5 out of 10 mice) than in MCS-exposed mice treated with either aspirin (2 out of 10 mice) or naproxen (one out of 10 mice). Conversely, irrespective of the administration of NSAIDs, almost all MCS-exposed mice exhibited the presence of microadenomas. As reported below, the histopathological findings were related to miRNA profiles in the serum and lung of each mouse.

#### Analysis of pulmonary and blood serum RNA

In the lung, the average amount of RNA extracted from the 40 specimens was 18.6 ± 1.06 μg. The overall 260/280 nm ratio was 2.0 ± 0.01, and the 260/230 nm ratio was 1.5 ± 0.06 (mean ± SE). The RNA amount obtained from the 40 blood serum samples was 0.3 ± 0.19 μg. The overall 260/280 absorbance ratio was 1.3 ± 0.05, whereas the 260/230 absorbance ratio could not be calculated because this parameter is not reliable for samples whose amounts are below the sensitivity threshold of nanophotometry. The presence of miRNA presence in the extracted samples was checked by capillary electrophoresis. The results indicated that miRNAs were adequately represented in the extracted samples, as highlighted by the presence of a peak in the 20 bp region, identified by comparison with the reference ladder (not shown).

#### Microarray analysis of pulmonary miRNAs

Microarray hybridization analysis of miRNA expression were performed in all lung samples belonging to each experimental group. The quality of this analysis was evaluated by calculating the call/response rate, which is the number of hybridized probes providing signals out of the total number of probes spotted on the microarray. The percentage of call/response rate was consistently >90%.

**Figure 7:**
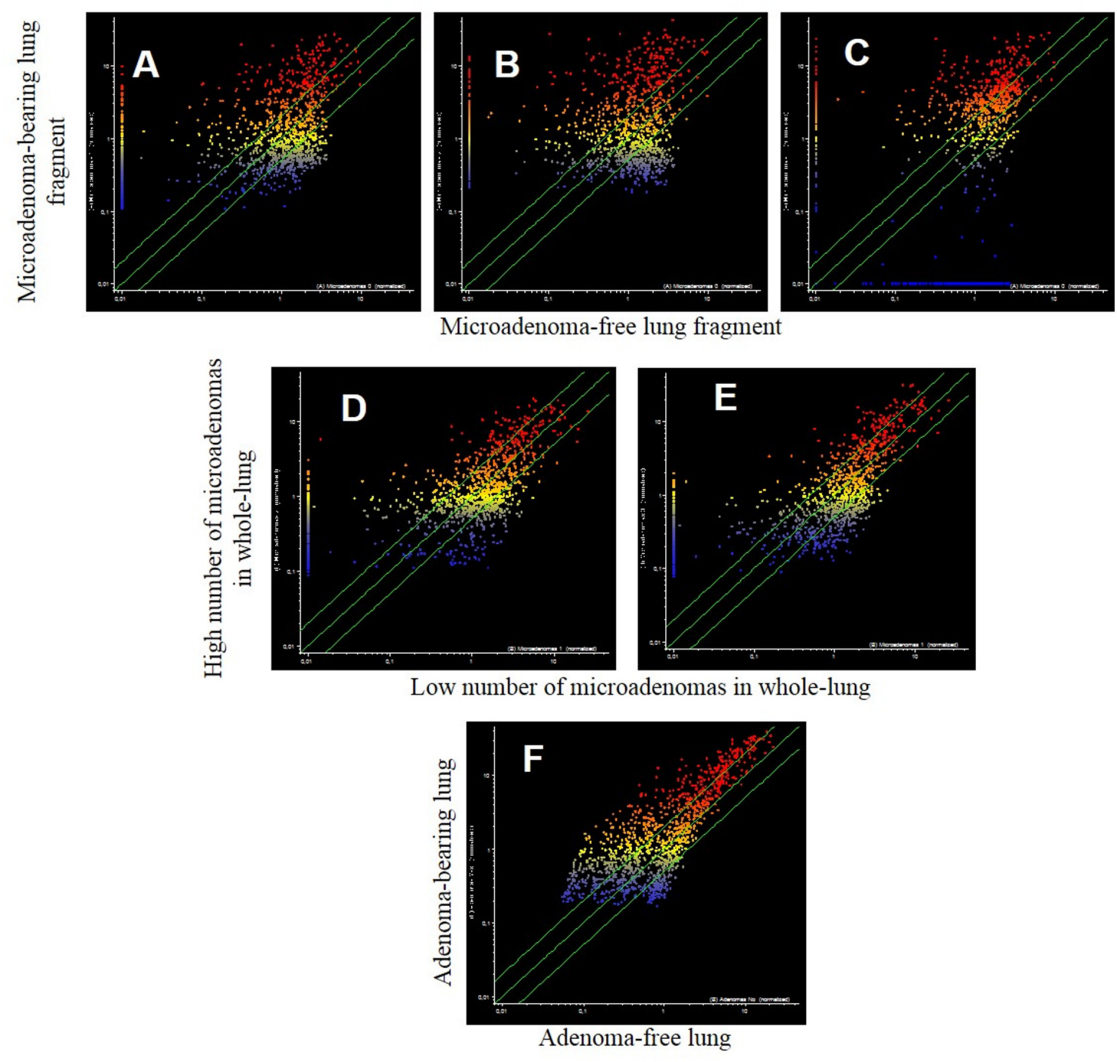
Scatter-plots comparing the expression of 1135 miRNAs in the lungs of 7.5-month old Swiss H mice of both genders exposed to MCS during the first 4 months of life and treated with aspirin after weaning *Upper panels*. miRNA expression was compared in the same lung fragments used for miRNA analysis between specimens without microadenomas (x-axis) or containing either 1-10 **(A)**, 11-20 **(B)** or >20 microadenomas **(C)** (y-axis). *Middle panels*. miRNA expression was compared in whole lungs between specimens containing 1-10 microadenoms (x-axis) with those containing either 11-20 microadenomas **(D)** or >20 microadenomas **(E)** (y-axis). *Bottom panel*. Comparison of miRNA expression as related to the presence of adenomas in the whole lung **(F).**

**Figure 8:**
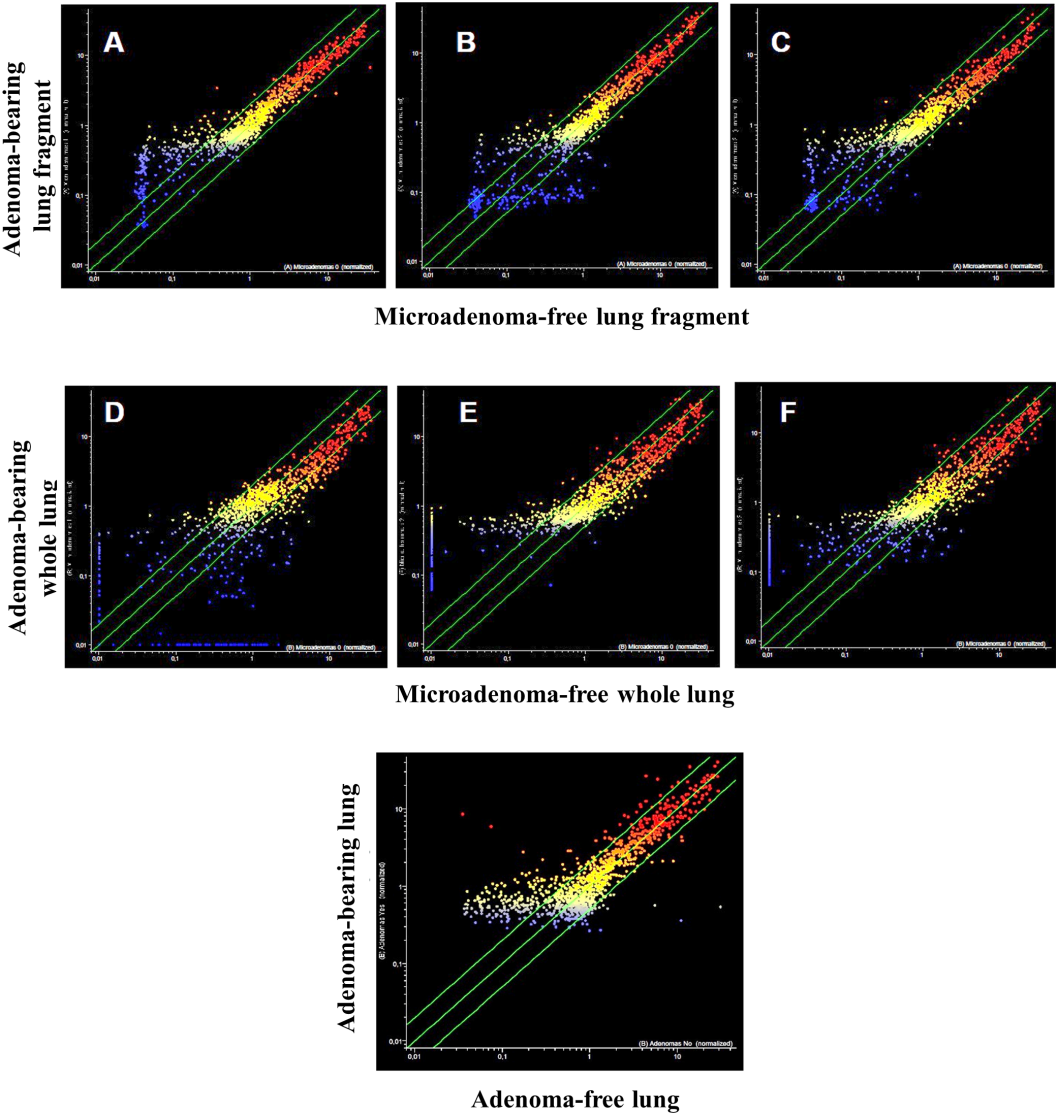
Scatter-plots analyzing the expression of 1135 miRNAs either in the lung fragments used for miRNA analysis (upper panels) or in the whole lungs (middle panels) of 7.5-month old Swiss H mice of both genders exposed to MCS during the first 4 months of life and treated with naproxen after weaning miRNAs were compared in mice without microadenomas (x-axis) vs. those containing either 1-10 **(A, D)**, 11-20 **(B, E)** or >20 microadenomas **(C, F)** (y-axis). The bottom panel refers to the comparison of miRNA expression in the whole lungs, either adenoma-free (x-axis) or bearing adenomas (y-axis).

The analysis of pulmonary miRNA expression profiles as related to the exposure of both male and female mice to MCS and to lung histology has previously been reported [27]. Further on, microarray data were processed by scatter-plot analysis to evaluate the efficacy of aspirin and naproxen in inhibiting MCS carcinogenicity by distinguishing between protected (cancer-free) and non-protected (cancer-bearing) mice. To that purpose, miRNA profiles were plotted in the MCS-exposed mice treated with aspirin by comparing the mice having a low number of microadenomas (<10) with those having higher numbers either of microadenomas (Figure 7, panels A-E) or adenomas (Figure 7, panel F). As inferred from the high number of miRNAs falling outside the 2-fold variation interval, miRNAs appear to be sensitive in identifying the mice protected by aspirin from MCS-related lung carcinogenesis as compared with those in which this chemopreventive drug failed to exert protective effects. Likewise, pulmonary miRNA profiles were compared in MCS-exposed mice treated with naproxen by distinguishing the mice free of microadenomas from those having higher numbers of microadenomas (Figure 8, upper and middle panels) and those bearing adenomas (Figure 8, bottom panel). As inferred from the high number of miRNAs falling outside the 2-fold variation interval in mice bearing either microadenomas or adenomas, miRNAs were quite sensitive in identifying the mice protected by naproxen from MCS-related carcinogenesis and those in which this chemopreventive drug did not exert protective effects.

**Figure 9:**
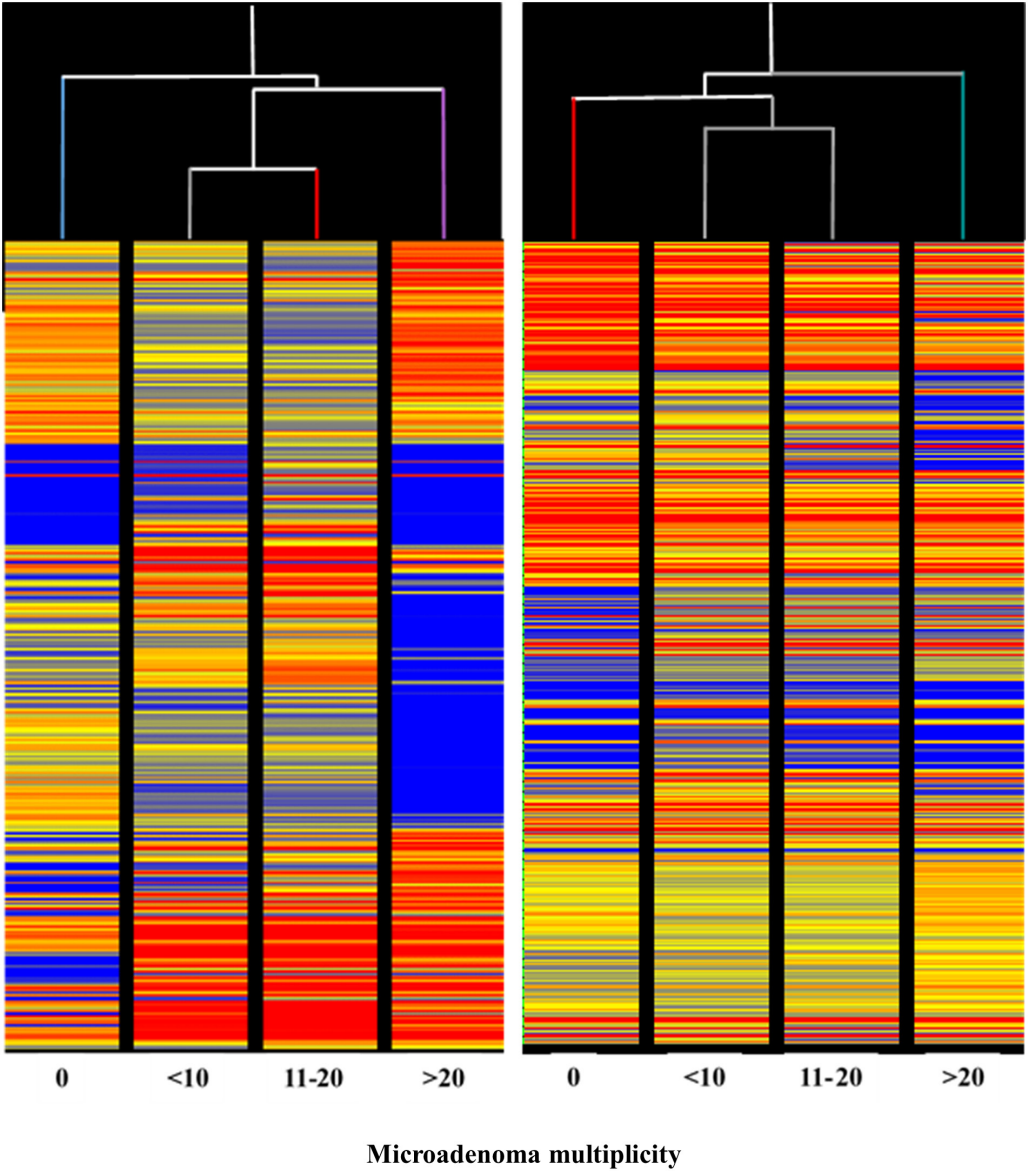
Unsupervised hierarchical cluster analysis (HCA) of the expression of 1135 miRNAs, according to the lung microadenoma multiplicity, in the lungs of 7.5-month old Swiss H mice of both genders exposed to MCS during the first 4 months of life and treated with either aspirin (left panel) or naproxen (right panel) after weaning

The HCA of miRNA profiles in the lung of mice receiving aspirin (Figure 9, left panel) indicated that miRNA expression is able to distinguish among lung specimens bearing varying numbers of microadenomas (i.e. 0, <10, 11-20, >20) induced by MCS. Indeed, this analysis showed that microadenoma–free mice cluster separately from other samples on the left in the hierarchical tree. Samples bearing intermediate numbers of microadenomas (i.e., <10 and 11-20) clustered together, whereas samples bearing high number of microadenomas (>20) clustered separately on the right in the dendrogram. The abundant blue color in the last column indicates that aspirin, at variance with other columns, failed to counteract miRNA downregulation induced by MCS in these specimens. Likewise, the HCA of miRNA profile in the lung of mice receiving naproxen (Figure 9, right panel) indicated that miRNA expression is able to distinguish among lung specimens bearing different numbers of microadenomas (i.e. 0, <10, 11-20, >20) induced by MCS. In fact, HCA showed that microadenoma–free mice clustered separately from the other samples. Samples bearing intermediate numbers of microadenomas (i.e., <10 and 11-20) clustered together, while samples bearing higher numbers of microadenomas clustered separately on the right in the hierarchical tree. In the last column, the blue color prevails as compared to other columns in the upper part, indicating that naproxen failed to counteract miRNA downregulation induced by MCS in these specimens for some miRNAs. More striking differences were observed by comparatively analyzing miRNA profiles in the lung of mice receiving either aspirin (Figure 7) or naproxen (Figure 8) according to the presence or absence of lung adenomas induced by MCS. HCA and PCA could not be performed in these cases because the samples were distributed according to a binomial class (adenoma yes/no), at variance with microadenomas that were distributed according to polynomial classes (0, <10, 11-20, >20).

**Figure 10:**
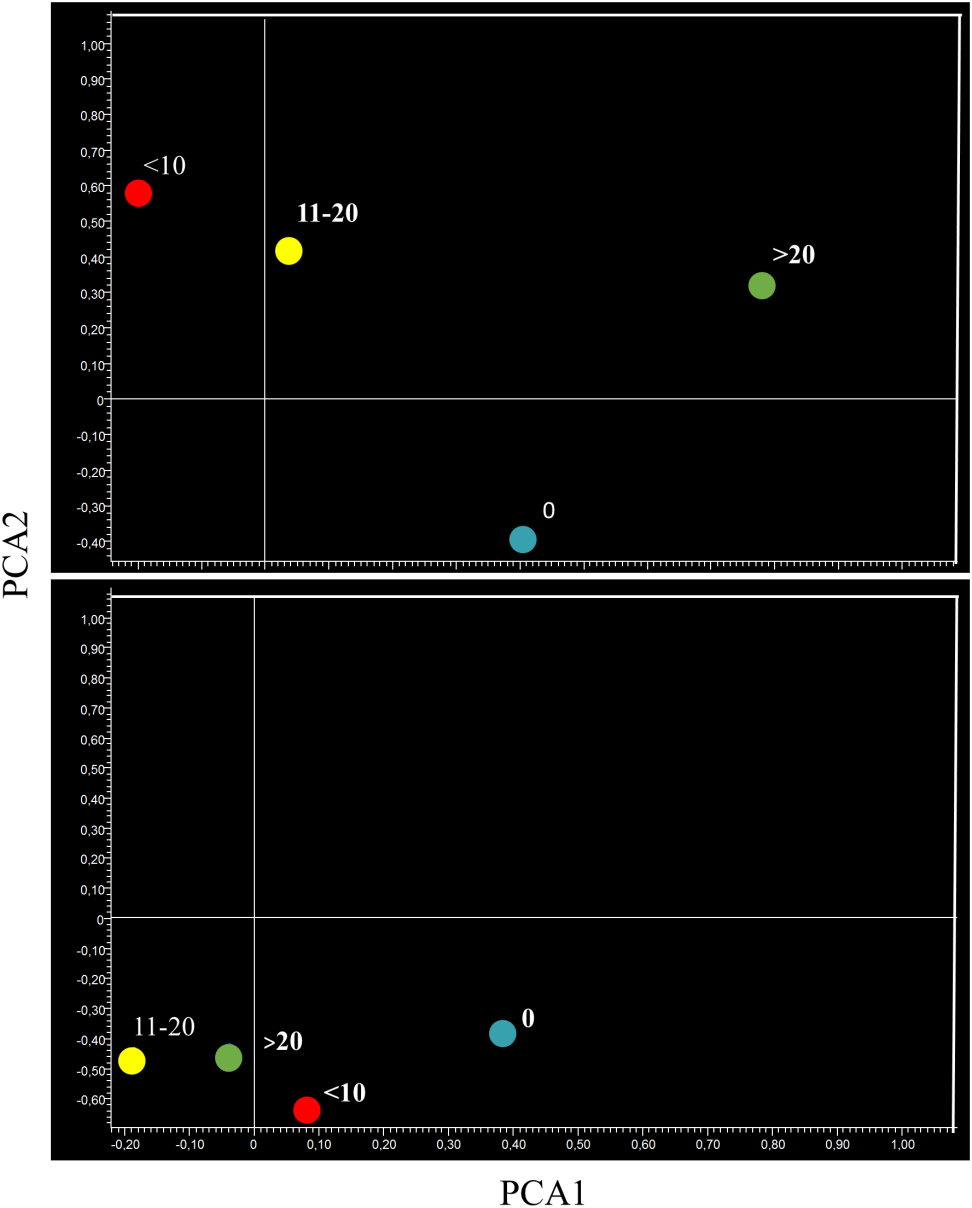
Bidimensional principal component analysis of variance (PCA) of the expression of 1135 miRNAs, according to the lung microadenoma multiplicity, in the lungs of 7.5-month old Swiss H mice of both genders exposed to MCS during the first 4 months of life and treated with either aspirin (upper panel) or naproxen (bottom panel) after weaning

PCA provided similar indications. In fact, the miRNA profiles of aspirin-treated mice bearing microadenomas were located separately from microadenoma-free mice in lung specimens (Figure 10, upper panel). Mice bearing 11-20 and >20 microadenomas fell in the same quadrant. However, the mice bearing intermediate number of microadenomas (11-20) were located in the left part of that quadrant, whereas mice bearing a high microadenoma multiplicity (>20) were located in the right part of it. As observed in lung specimens of mice treated with naproxen (Figure 10, bottom panel), the mice free of adenomas and those bearing <10 microadenomas were located in the same quadrant, which was different form the quadrant encompassing mice bearing either 11-20 or >20 microadenomas.

The miRNA changes reported in Tables 5–8 were either in the sense of downregulation (fold variations <1.0) or upregulation (fold variations >1.0). A general trend towards downregulation prevailed in microadenoma- and adenoma-bearing mice as compared to lesion-free mice (Tables 5 and 6).

#### Microarray analysis of blood serum miRNAs

Despite the very low quantity of samples available, microarray hybridization of blood serum miRNA was of high quality, the call response rate being >90%, and the balance between the intensity signal recorded from the two channels (Cy3/Cy5) was optimal (data not shown).

**Figure 11:**
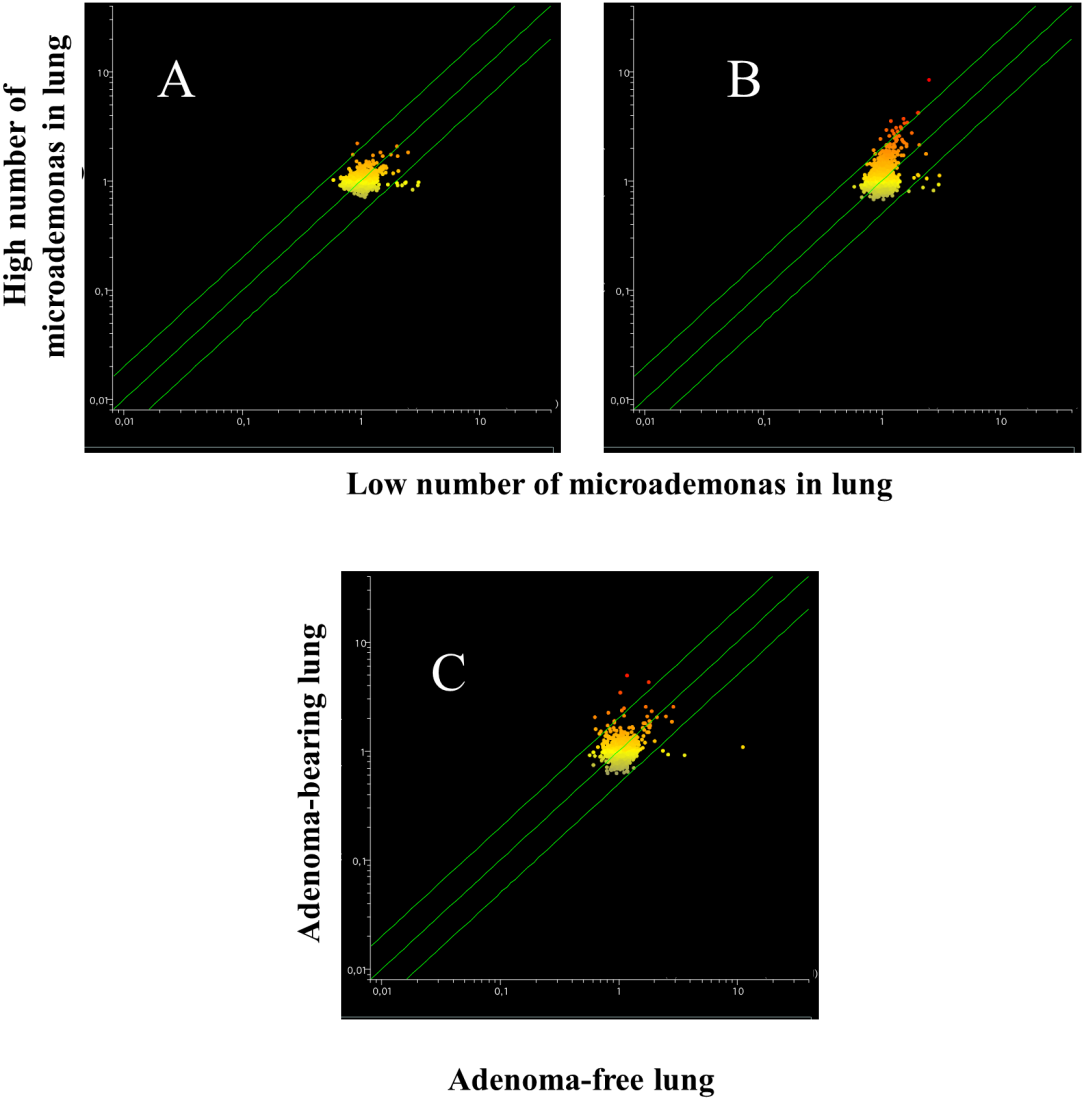
Scatter-plots comparing the profiles of 1135 miRNAs in the blood serum of 7.5-month old Swiss H mice of both genders exposed to MCS during the first 4 months of life and treated with aspirin after weaning, as related to the presence either of microadenomas (upper panels) or adenomas (bottom panel) *Upper panels*. miRNA expression was compared between specimens containing 1-10 microadenomas (x- axis) with those containing either 11-20 microadenomas **(A)** or >20 microadenomas **(B)** (y-axis). *Bottom panel*. Comparison of miRNA expression in blood serum as related to the presence of adenomas in lung from aspirin-treated mice **(C)**.

**Figure 12:**
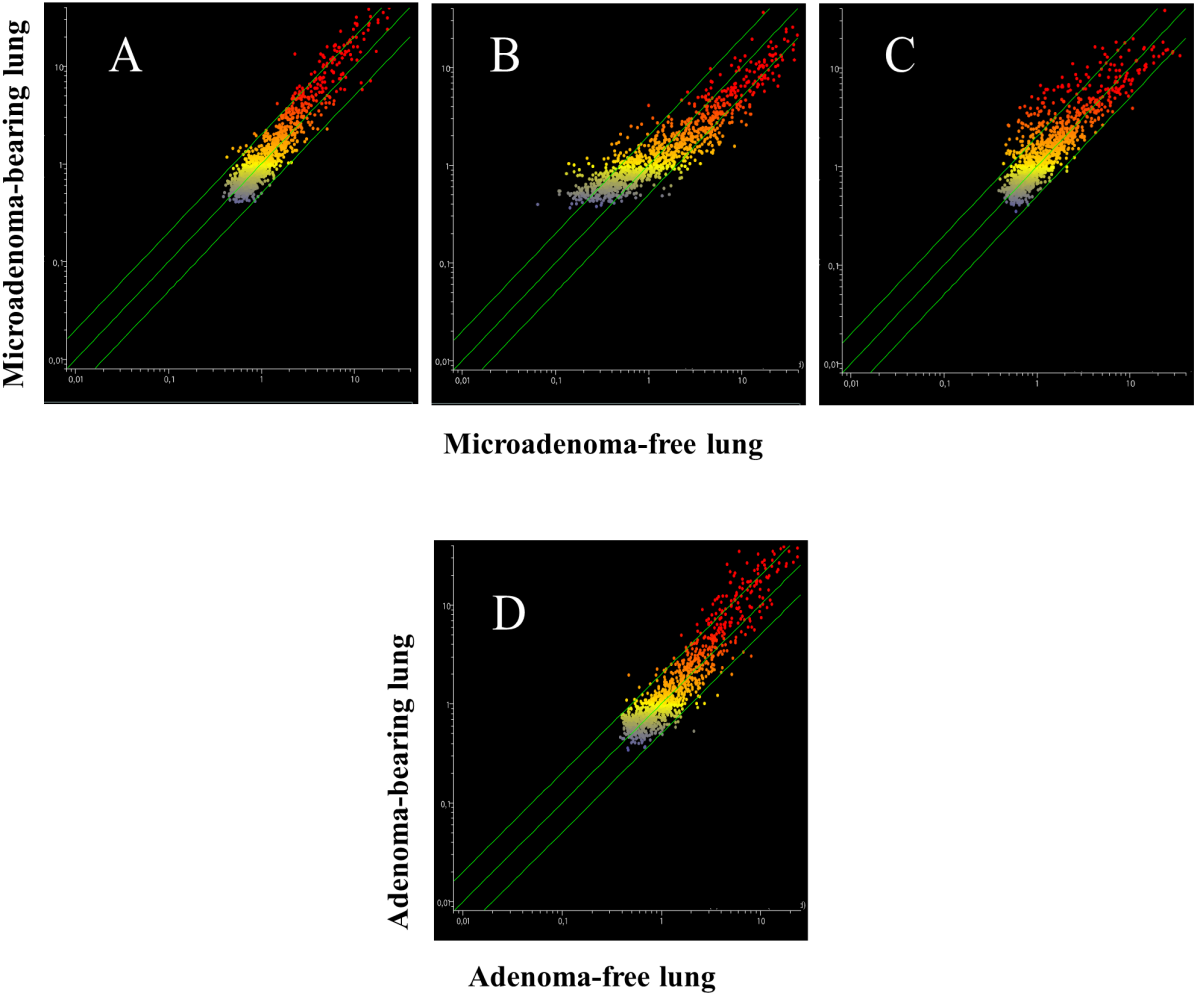
Scatter-plots comparing the profiles of 1135 miRNAs in the blood serum of 7.5-month old Swiss H mice of both genders exposed to MCS during the first 4 months of life and treated with naproxen after weaning, as related to the presence either of microadenomas (upper panels) or adenomas (bottom panel) *Upper panels.* Blood serum miRNAs were compared in mice without lung microadenomas (x- axis) versus those containing either 1-10 **(A)**, 11-20 **(B)** or >20 microadenomas **(C)** (y-axis). *Bottom panel* **(D).** Scatter-plot comparing miRNA expression in the blood serum of MCS-exposed mice treated with naproxen either without adenomas (x-axis) or bearing adenomas in the whole lung (y-axis).

Blood serum microarray data were analyzed to evaluate the efficacy of aspirin and naproxen in inhibiting MCS-related carcinogenesis. To this purpose, miRNA profiles were compared by scatter-plot analyses in the blood serum of MCS-exposed mice treated with aspirin by distinguishing the mice having a low number of microadenomas (<10) from those having higher numbers either of microadenomas (Figure 11, upper panels A and B) or of adenomas (Figure 11, bottom panel). As inferred from the miRNAs falling outside the 2-fold variation interval, it appears that blood miRNAs can contribute to identify the mice protected by aspirin against MCS-related carcinogenesis as compared to those in which this chemopreventive drug failed to exert protective effects. Comparison of blood miRNA profiles in MCS-exposed mice treated with naproxen was made by distinguishing the mice free of microadenomas from those having higher numbers of microadenomas (Figure 12, panels A-C) and those bearing adenomas (Figure 12, panel D). As inferred from the miRNAs falling outside the 2-fold variation interval in mice with either microadenomas or adenomas, it appears that miRNAs can contribute to distinguish the mice protected by naproxen from MCS carcinogenesis from those in which this chemopreventive drug did not exert protective effects. By comparing the number of miRNAs located outside the 2-fold variation interval (Figures 11 and 12), blood miRNAs seem to be more sensitive in identifying the mice protected by naproxen than those protected by aspirin.

**Figure 13:**
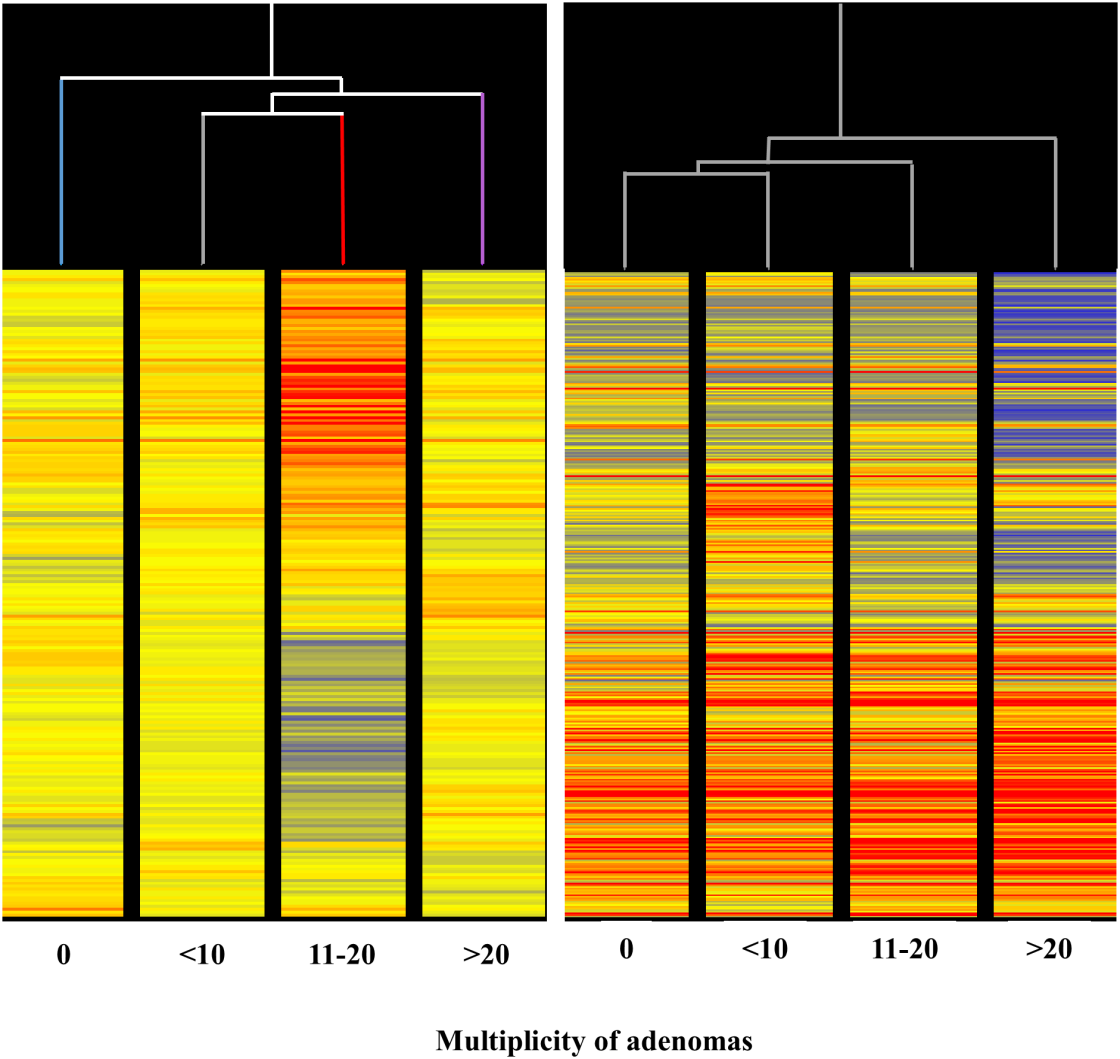
Unsupervised hierarchical cluster analysis (HCA) of the profiles of 1135 miRNAs, according to the lung microadenoma multiplicity, in the blood serum of 7.5-month old Swiss H mice of both genders exposed to MCS during the first 4 months of life and treated with either aspirin (left panel) or naproxen (right panel) after weaning

HCA of circulating miRNAs in aspirin-treated mice (Figure 13, left panel) showed a sample distribution in the hierarchical tree similar to the one described for pulmonary miRNAs. However, the differences in blood serum were less striking than in lung, and the presence of a blue color (reflecting miRNA downregulation) was not clearly distinguishable. At variance with the situation in the lung, naproxen-treated mice bearing <10 and 11-20 microadenomas clustered together with microadenoma-free mice. Only the mice bearing >20 microadenomas were located separately in the right part of the dendrogram (Figure 13, right panel).

**Figure 14:**
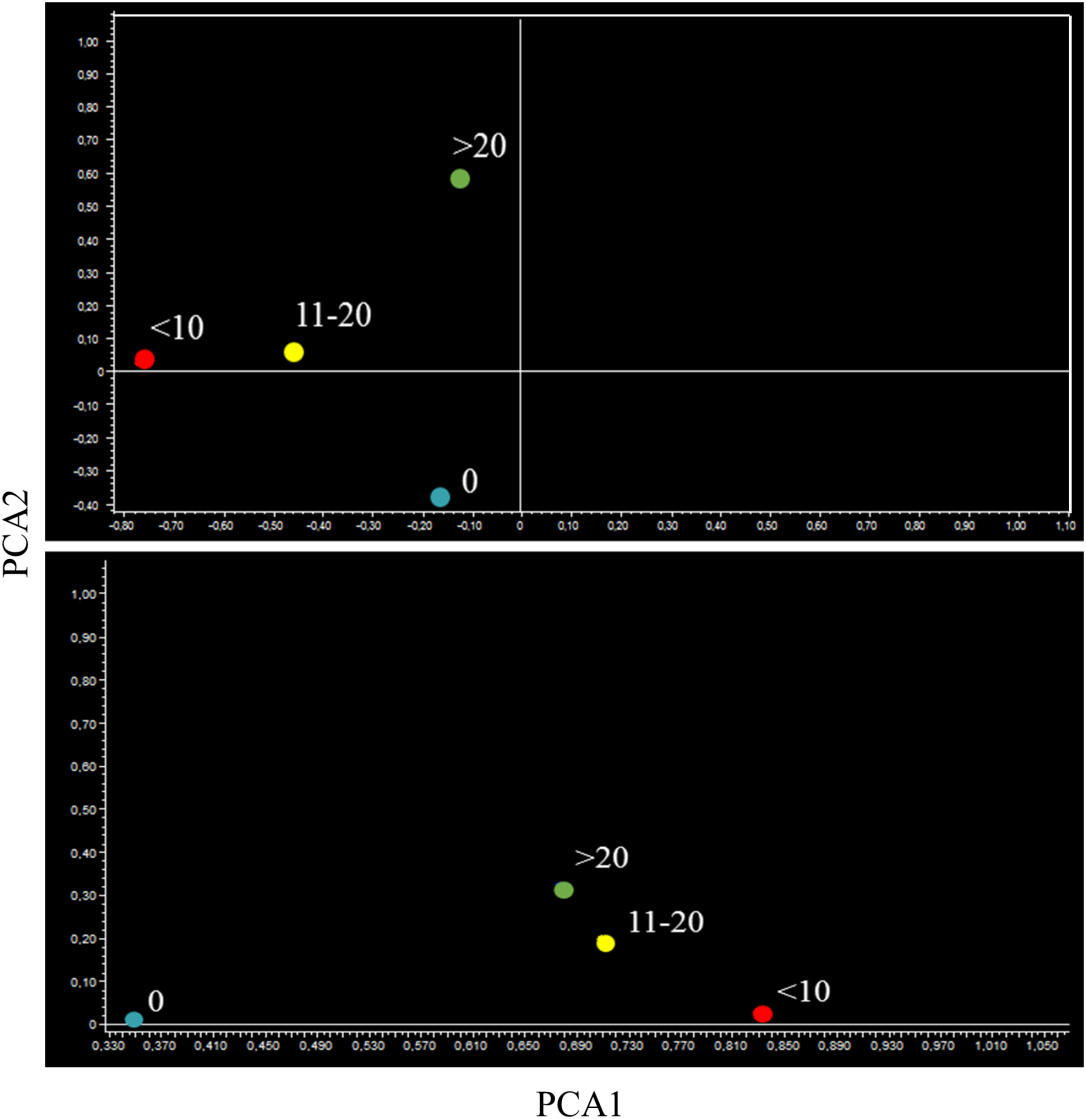
Bidimensional principal component analysis of variance (PCA) of the overall profiles of 1135 miRNAs, according to the lung microadenoma multiplicity, in the blood serum of 7.5-month old Swiss H mice of both genders exposed to MCS during the first 4 months of life and treated with either aspirin (upper panel) or naproxen (bottom panel) after weaning

When analyzing miRNA expression in blood serum by PCA, the microadenoma-free samples in aspirin-treated mice were located in a different quadrant from microadenoma-bearing mice, irrespective of their multiplicity (Figure 14, upper panel). In the blood of naproxen-treated mice, all symbols fell in the same quadrant. However, microadenoma-free mice were located on the left, far from microdenoma-bearing mice that were located on the right of the same quadrant, irrespective of their multiplicity (Figure 14, lower panel).

### Identification of miRNAs modulated by either aspirin or naproxen in mouse lung and blood serum

The differentially modulated miRNAs by the two NSAIDs in mice bearing <10 or >10 microadenomas and/or free or bearing adenomas (adenoma yes or no) were identified by volcano plot analyses both in lung and blood serum (not shown). These miRNAs and their main functions are listed in Tables 5 and 6 for aspirin and in Tables 7 and 8 for naproxen, respectively. Aspirin and naproxen upregulated some miRNAs involved in protective functions, such as inhibition of inflammation or apoptosis.

## DISCUSSION

The two studies described in the present paper had the common goal of evaluating the expression profiles of 1135 miRNAs in both lung and blood serum of mice exposed to CS since birth and/or treated with NSAIDs. Transcriptomics data are poorly informative of the biological events occurring at the phenotypic level during the carcinogenesis process, because the 97% of messenger RNA does not the ribosomes, being mainly destroyed by miRNA scavenging [28]. This circumstance emphazises the importance of using miRNAs as biomarkers of both safety and efficacy of cancer chemopreventive agents. The doses of aspirin and naproxen used in the present study were 3–4 times higher than those that are commonly used in humans for therapeutic purposes. However, these doses, which had been chosen based on a preliminary subchronic toxicity test, did not cause the presence of occult blood in mouse feces [26] and in the long term they did not produce any appreciable adverse effects in parallel cancer chemoprevention studies [21, 26]. In addition, as to the translatability of mouse data to the human situation, it should be kept in mind that the oral intake with the diet is highly fractionated during the day, because mice eat continuously, depending on their metabolic needs.

The two studies were different in their objectives for several reasons. In fact, the first one used mice of the A/J strain, which is considered to be the most sensitive strain to pulmonary carcinogens and has been used in studies that evaluated the tumorigenicity of ECS [22]. Conversely, the second study used mice of the Swiss H strain, which has been shown to be sensitive to the induction [19, 20] and modulation [8] of lung tumors induced by MCS. For technical reasons, the TPM concentration present in MCS was higher than that present in ECS, which in principle is acceptable because MCS is inhaled as an undiluted complex mixture by active smokers, whereas ECS is inhaled after dilution in ambient air. Although the ECS concentrations used were high, they were compatible with those measured in certain indoor environments [3]. A crucial difference between the two studies was that the analysis of miRNAs was made at 10 weeks of age in A/J mice exposed to ECS and at 7.5 months of age in Swiss H mice exposed to MCS.

Accordingly, evaluation of miRNA profiles in A/J mice exposed to ECS and/or receiving either aspirin or naproxen was made at an early time, when no histopathological lesion was detectable in mouse lung, although nucleotide alterations were already evident [26]. Therefore, such a situation may mimic a pharmacological intervention in subjects, either unexposed or exposed to passive smoking since birth, in whom an early diagnosis is only feasible by means of molecular tools in a secondary prevention setting.

At the above doses, the two drugs, and especially naproxen, caused some dysregulation of the baseline expression of miRNAs in the lung of smoke-free A/J mice. In fact, aspirin and naproxen altered the expression of 0.6% and 1.5% of the analyzed pulmonary miRNAs, respectively. Consistent with the mechanisms of action of these NSAIDs, aspirin dysregulated one miRNA involved in COX-2 regulation and one miRNA involved in inflammation, whereas naproxen dysregulated 4 miRNAs involved in COX regulation, one miRNA involved in prostaglandin activation, and 3 miRNAs involved in inflammation. Other miRNAs were dysregulated as well, showing that these drugs have composite mechanisms of action. A few miRNAs showed altered levels also in the blood serum of smoke-free mice treated with aspirin and especially with naproxen. However, as discussed below, these miRNAs were different from those that were dysregulated in the lung, and were involved in miscellaneous functions other than regulation of COX activity and of inflammation.

Exposure of mice to ECS, in the absence of NSAIDs, caused an extensive dysregulation of pulmonary miRNAs, mainly in the sense of downregulation. These results are in line with those observed in other rodents exposed to the same complex mixture, including neonatal ICR(CD-1) mice exposed for 5-6 weeks [29, 30] and adult Sprague-Dawley rats exposed for 4 weeks [31]. ECS-downregulated miRNAs included both miRNAs that are involved in adaptive functions, protecting the lung from noxious ECS components, and miRNAs that are involved in the pathogenesis of smoke-related diseases. Administration of NSAIDs tended to counteract the ECS-related dysregulation of the global miRNA expression profiles in mouse lung by approaching, especially in the case of naproxen-treated mice, the scenario observed in sham-exposed mice. Both agents attenuated the ECS-related alterations of miRNAs involved in inflammation and cell proliferation and, in the case of aspirin, one miRNA involved in COX regulation. These data highlight the fact that NSAIDs work not only through anti-inflammatory and antioxidant mechanisms via the COX pathway but also through other mechanisms, and support the view that these drugs possess antiproliferative properties that play a role in cancer prevention. In fact, COX-2 overexpresssion promotes cell proliferation by increasing the levels of prostaglandins [32], and additionally aspirin has been shown to induce antiproliferative effects by non-prostaglandin-dependent pathways [33].

Both aspirin and naproxen, irrespective of gender, mitigated the formation of bulky DNA adducts and oxidative DNA damage in the same lung samples used in the present study for miRNA analysis [26]. However, at 9 months of age, the NSAIDs selectively inhibited the ECS-induced formation of lung adenomas in female A/J mice only [26]. A similar selective inhibition of lung tumors was observed in MCS-exposed female Swiss H mice [21], in which 3 miRNAs (*miR-10a*, *miR-125*, and *miR-130a*) involved in estrogen and HER2 pathways were differentially expressed in adenoma-bearing male and female mice [27]. These findings, which support a role for estrogens in smoke-related pulmonary carcinogenesis and the involvement of epigenetic mechanisms in the protective properties of NSAIDs, prompted us to explore intergender differences in pulmonary miRNA profiles as related to exposure to ECS and/or treatment with either aspirin or naproxen. This part of the study highlighted a complex situation and showed that many miRNAs were differentially expressed in males and females belonging to all experimental groups. Some of them, such as *miR-30b*, *miR-92a*, and *miR-125b*, have been reported to undergo intergender differences in expression during lung development [34]. Studies in mice showed that tobacco smoke can stimulate the metabolism of estrogens in the lung, and estrogen metabolites could potentially contribute to lung tumor development [35, 36]. Mainly based on our previous studies, it has been postulated that elevated levels of estrogens or their metabolites in the lungs of CS-exposed rodents cause the observed CS-induced downregulation of miRNAs [37]. Estrogens, via estrogen receptors, modulate airway and lung functioning [38] and, like other nuclear receptors, estrogen receptors can regulate miRNA expression [39]. On the other hand, NSAIDs have antiestrogenic properties, which are due to the fact that prostaglandin E_2_ upregulates the expression of the aromatase gene (aromatase CYP), the product of CYP19, which catalyzes the final step in estrogen biosynthesis [40].

As to the second study reported in the present paper, evaluation of miRNA expression profiles in Swiss H mice aged 7.5 months mimicked an intervention in which the NSAIDs are administered from early adulthood to the end of the experiment. In that case, changes in miRNA profiles can be interpreted either as changes persisting 3.5 months after discontinuation of exposure to MCS and/or to miRNA alterations associated with the development of histopathological lesions. We previously reported and discussed the effects of MCS on pulmonary and blood serum miRNA profiles as related to the occurrence of preneoplastic and neoplastic lesions in mouse lung at 7.5 months of age [27]. Again, these alterations were affected by the mouse gender consistent with the findings that, at the same time, aspirin and even more sharply naproxen inhibited the formation of MCS-induced lung tumors in female mice only [27]. In addition, at 4 months of life, naproxen attenuated the systemic genotoxic damage in female mice exposed to MCS [21].

Here we evaluated the ability of aspirin and naproxen to modulate miRNA alterations as related to their protective effects against the MCS-induced formation of pulmonary microadenomas and adenomas at 7.5 months of age. Overall analyses provided evidence for the differential expression of pulmonary miRNAs in the mice in which the two NSAIDs inhibited the occurrence of these histopathological lesions. In particular, some of the miRNAs modulated by aspirin in mice protected against the formation of microadenomas (*miR-16*, *miR-133*, *miR-137*, and *miR-191*) were the same that had been found to be modulated by the same NSAID in A/J mice aged 10 weeks. These miRNAs are involved in COX modulation, inflammation, cell proliferation and apoptosis. Most of the other miRNAs distinguishing the mice according to the yield of microadenomas (*miR-30*, *miR-181b*, *miR-183*, *miR-301a*, *miR-350*, *miR-466a*, and *miR-466i*) were also able to distinguish the mice according to the yield of adenomas. This category of miRNAs is involved in mechanisms affecting later stages of pulmonary carcinogenesis, such as stem cell recruitment, intercellular adhesion, and multidrug resistance. Similar patterns were observed in naproxen-treated mice. In fact, a couple of miRNAs (*miR-27a* and *miR-133a*), targeting inflammation and cell proliferation, had been found to be modulated by the same NSAID in A/J mice aged 10 weeks, whereas other miRNAs (*miR-30*, *miR-101* and *miR-344b*) affecting later stages of pulmonary carcinogenesis were able to distinguish the mice according to the yield of both microadenomas and adenomas. Two of the above mentioned miRNAs (*miR-30* and *miR-133*) were targeted by both aspirin and naproxen.

In the present study, blood serum miRNAs were analyzed in order to evaluate the efficacy of NSAIDs to interfere in MCS-related carcinogenesis. Like pulmonary miRNAs, circulating miRNAs were able to distinguish the mice protected by aspirin and naproxen against the induction of microadenomas and adenomas. One miRNA only (*miR-466*) was altered in both blood serum and lung of mice with a high yield of microadenomas. Conversely, 13 miRNAs were altered in both body compartments of mice bearing adenomas. In these mice, 5 miRNAs were altered in blood but not in lung (*miR-34b*, *miR-106a*, *miR-449*, *miR-466*, *miR-493*). In aspirin-treated mice exposed to MCS, modulation of 9 miRNAs in both lung and blood serum (*miR-30c*, *miR-181b*, *miR-183*, *miR-301a*, *miR-350*, *miR-466a/i*, *miR-500*, and *miR-709*) correlated with protection against pulmonary microadenomas, while no miRNA related to protection against pulmonary adenomas was modulated at the same time in both body compartments. Likewise, in naproxen-treated mice exposed to MCS modulation of 3 miRNAs in both lung and blood serum (*miR-181b*, *miR-344d*, and *miR-708*) correlated with protection against pulmonary microadenomas, while one miRNA only (*miR-*711), correlated with protection against pulmonary adenomas, was modulated in both body compartments. These findings indicate that the blood miRNA signature is more informative about the chemopreventive efficacy of NSAIDs when analyzed during early steps of the carcinogenesis process.

In conclusion, the results emerging from the two studies reported in the present paper confirm that the analysis of pulmonary miRNAs is a sensitive molecular tool to detect the disrupting effects of smoke exposure to either MCS or ECS. In addition, they also showed the ability of NSAIDs to modulate both early and late stages of pulmonary carcinogenesis. The role of blood serum miRNAs in smoke-related carcinogenesis and in cancer chemoprevention by NSAIDs is more uncertain, presumably because circulating miRNAs reflect the contribution from multiple organs and not only from the lung. Such an interpretation is supported by the indications of a further study in which we are evaluating miRNA expression profiles in 3 biological fluids (blood serum, bronchoalveolar lavage fluid, and urines) and 10 organs of young Swiss ICR(CD-1) mice exposed to MCS for 2 months and/or treated with the selective COX-2 inhibitor celecoxib. The preliminary results suggest that the profiles of miRNAs are affected by pharmacokinetic features, since blood and urine receive miRNA contributions from multiple organs, mainly including skeletal muscle, liver, and kidney, whereas bronchoalveolar lavage mainly receives miRNAs from lung.

## MATERIALS AND METHODS

### Aspirin and naproxen

Aspirin and naproxen were purchased from Sigma-Aldrich (Milan, Italy). A daily oral dose of 1600 mg/kg diet for aspirin and 320 mg/kg diet for naproxen was used in both miRNA studies, based on the results of a subchronic toxicity study in post-weanling A/J mice [26], on literature data relative to experimental studies in mice, and taking into account the therapeutic doses used in humans.

### Evaluation of early effects of aspirin and naproxen on miRNA profiles in the lung and blood of A/J mice, either unexposed or exposed to ECS

#### Mice

A/J mice were born in the laboratory in Genoa (Italy) from males and females purchased from Harlan Laboratories (San Pietro al Natisone, Udine, Italy). The mice were housed in MakrolonTM cages on sawdust bedding and maintained on standard rodent chow (Teklad 9607, Harlan Laboratories) and tap water *ad libitum*. The animal room temperature was 23 ± 2°C, with a relative humidity of 55% and a 12-h day-night cycle. Housing and treatment of mice were in accordance with NIH, European (2010/63/UE Directive), and institutional guidelines.

#### Treatment of mice

For the analysis of intermediate biomarkers, including miRNA expression profiles, newborn mice were divided into six groups, each one composed of 5 males and 5 females including (*a*) mice kept in filtered air for 10 weeks (sham-exposed mice); (*b*) mice receiving a diet supplemented with aspirin, starting after weaning (∼4 weeks) and continuing for an additional 6 weeks; (*c*) mice receiving a diet supplemented with naproxen, starting after weaning (∼4 weeks) and continuing for an additional 6 weeks; (*d*) mice exposed to ECS for 10 weeks, starting within 12 h after birth (ECS-exposed mice); (*e*) ECS-exposed mice receiving a diet supplemented with aspirin, starting after weaning (∼4 weeks) and continuing for an additional 6 weeks; (*f*) ECS-exposed mice receiving a diet supplemented with aspirin, starting after weaning (∼4 weeks) and continuing for an additional 6 weeks.

#### Exposure of mice to ECS

A whole-body exposure of mice to ECS was achieved by using a smoking machine (model TE-10C, Teague Enterprises, Davis, CA) mixing MCS (11%) and sidestream CS (89%) generated by burning at one time 3R4F Kentucky reference cigarettes (College of Agriculture, The Reference Cigarette Program, University of Kentucky, Lexington, KY), having a declared content of 9.4 mg tar and 0.7 mg nicotine and delivering 12 mg CO each. The FTC (Federal Trade Commission) method of puffing for 2 s, once a min, at a volume of 35 cm^3^ was used. Two rounds of exposure were performed daily by burning a total of 120 cigarettes per day. The total particulate matter in the exposure chambers was on an average 95 mg/m^3^ and CO was 610 ppm.

#### Collection of biological samples for the analysis of miRNA expression

At the age of 10 weeks, all mice were euthanized by following the 2013 AVMA guidelines on euthanasia using slow introduction of CO_2_ asphyxiation. Death was confirmed by absence of respiration and/or heartbeat. Blood was immediately collected by heart puncture and used for preparing serum. The whole lungs were collected individually and immersed in RNAlater (Qiagen, Valencia, CA).

### Evaluation of late effects of aspirin and naproxen on miRNA profiles in the lung and blood of Swiss H mice, either unexposed or exposed to MCS

#### Mice

Strain H neonatal mice were born at the Animal Laboratory of the National Center of Oncology (Sofia, Bulgaria). The mice were housed in Makrolon™ cages on sawdust bedding and maintained on standard rodent chow (Kostinbrod, Bulgaria) and allowed drinking water ad libitum. Housing, breeding, and treatment of mice were in accordance with NIH and European (86/609/EEC Directive) guidelines.

#### Treatment of mice

The neonatal mice were randomized and divided into four experimental groups, including (*g*) untreated mice kept in filtered air for 7.5 months (sham-exposed mice); (*h*) mice exposed to MCS, starting within 12 h after birth and continuing daily during the first 4 months of life, followed by an additional 3.5 months in filtered air (MCS-exposed mice), (*i*) MCS-exposed mice receiving aspirin with the diet, starting after weaning and continuing until the end of the experiment, and (*j*) MCS-exposed mice receiving naproxen with the diet, starting after weaning and continuing until the end of the experiment.

#### Exposure of mice to MCS

The mice were exposed whole-body to the MCS generated by commercially available cigarettes (Melnik King Size, Bulgartabac), having a declared content of 9.0 mg tar and 0.8 mg nicotine and delivering 10 mg CO each. MCS was delivered to the exposure chambers by drawing 15 consecutive puffs, each of 60 ml and lasting 6 s. Each daily session involved six consecutive exposures, lasting 10 min each, with 1-min intervals, during which a total air change was made. The average concentration of total particulate matter in the exposure chambers was 527 mg/m^3^.

#### Collection of biological samples for the analysis of miRNA expression

A subset of 40 mice (20 males and 20 females), randomized from broader groups of mice used for evaluating the systemic genotoxic damage after 4 months and tumors as well as other histopathological alterations after 7.5 months [21], were used for assessing miRNA expression profiles. After euthanasia using slow introduction of CO_2_ asphyxiation, blood was immediately collected by heart puncture and used for preparing serum. The whole lungs were collected, divided into 10 sections and used for histopathological analysis. In particular, the accessory, middle, and caudal lobes of the right lung were cut into two pieces each, whereas the cranial lobe was left uncut. The left lung was cut into 3 pieces. A fragment of the right caudal lobe was divided into two parts, one fixed in 10% formalin and used for histopathological analysis (1 section/mouse) and the other one immersed in RNAlater (Qiagen, Valencia, CA) and used for miRNA analysis.

### Evaluation of miRNAs in lung fragments and blood serum

#### miRNA extraction

The lung fragments (10 mg each) were homogenized in QIAzol Lysis Reagent (700 μl) by continuous shaking in Tissue Lyser (Qiagen) for 2 min at 30 Hz. The homogenates were centrifuged at 14000 x *g* at 4°C for 15 min to remove cell debris, and pulmonary miRNAs were purified from the supernatants by using the miRNeasy kit (Qiagen). Blood serum miRNA was isolated by using the Exiqon’s miRCURY™ RNA Isolation Kit from Biofluids (Exiqon, Vedbaek, Denmark). The amount and purity of extracted RNA were evaluated by fiber optic spectrophotometer (Nanodrop ND-1000), and the 230/260 and 260/280 absorbance ratios were calculated. The RNA structural integrity (RIN) was evaluated by capillary electrophoresis using a RNA bioanalyzer (Bioanalyzer Agilent 2100, Agilent Santa Clara, CA) equipped with a RNA oligonucleotide chip (RNA 6000 Nano Ladder Chip, Agilent). The miRNA amounts were standardized among blood serum samples for microarray and qPCR analyses using Qubit™ 3.0 Fluorometer (Life Technologies, Gent, Belgium).

### miRNA expression analysis

miRNA expression analysis in all individual lung and blood serum specimens was carried out by microarrays (miRCURY LNA™ microRNA Array, Exiqon) containing 3100 capture probes covering human, mouse and rat miRNAs. In particular, this microarray analyzes the expression of 1135 mouse miRNAs. The RNA from each sample was labeled with Label IT^®^ miRNA Labeling Kits, Version 2 (Mirus Bio, WI) following the standard protocol. Total RNA (500 ng) was mixed with 10 μl of 10x labeling buffer, 4 μl Label IT reagent (containing Cy 3 or Cy 5 fluorescent tracers), and water to 86 μl. The samples were incubated at 36°C for 1 h and the reaction was stopped by adding 10 μl Stop Reagent. The samples were purified onto a chromatographic column, and hybridized to the microarray in GlassArray Hybridization Cassettes (Invitrogen Ltd, Paisley, UK) in a water bath at 37°C for 16 h. After a wash sequence, the microarrays were dried by centrifugation and scanned by laser scanner (ScanArray, PerkinElmer, Waltham, MA).

Microarray data were validated by real time-qPCR for *miR*-*146a*, *miR-191*, *miR-199b*, and *miR-223* as previously described [27].

### Statistical analysis

After local background subtraction, log transformation and normalization miRNA microarray data were analyzed by GeneSpring software (Agilent, Santa Clara, CA), and expression data were median centered by using the GeneSpring normalization option. Comparisons between sets of data were done by evaluating the fold variations of duplicate data generated for each miRNA. In addition, the statistical significance of the differences was evaluated by means of the GeneSpring ANOVA applied by using Bonferroni multiple testing correction. As inferred from volcano-plot analysis, differences between sets of data were taken as significant when they were statistically significant (P < 0.05) and showed >2-fold variations. qPCR data were expressed as means ± SD of 3 replicates, and differences between groups were evaluated by Student’s *t* test for unpaired data.
